# A PPIX-binding probe facilitates discovery of PPIX-induced cell death modulation by peroxiredoxin

**DOI:** 10.1038/s42003-023-05024-5

**Published:** 2023-06-24

**Authors:** John Lynch, Yao Wang, Yuxin Li, Kanisha Kavdia, Yu Fukuda, Sabina Ranjit, Camenzind G. Robinson, Christy R. Grace, Youlin Xia, Junmin Peng, John D. Schuetz

**Affiliations:** 1 Department of Pharmaceutical Sciences, St. Jude Children’s Research Hospital, 262 Danny Thomas Place, Memphis, TN 38105 USA; 2 Department of Structural Biology, St. Jude Children’s Research Hospital, 262 Danny Thomas Place, Memphis, TN 38105 USA; 3 Center for Proteomics and Metabolomics, St. Jude Children’s Research Hospital, 262 Danny Thomas Place, Memphis, TN 38105 USA; 4 Cellular Imaging Shared Resource, St. Jude Children’s Research Hospital, 262 Danny Thomas Place, Memphis, TN 38105 USA

**Keywords:** Molecular medicine, Biochemistry, Chemical biology

## Abstract

While heme synthesis requires the formation of a potentially lethal intermediate, protoporphyrin IX (PPIX), surprisingly little is known about the mechanism of its toxicity, aside from its phototoxicity. The cellular protein interactions of PPIX might provide insight into modulators of PPIX-induced cell death. Here we report the development of PPB, a biotin-conjugated, PPIX-probe that captures proteins capable of interacting with PPIX. Quantitative proteomics in a diverse panel of mammalian cell lines reveal a high degree of concordance for PPB-interacting proteins identified for each cell line. Most differences are quantitative, despite marked differences in PPIX formation and sensitivity. Pathway and quantitative difference analysis indicate that iron and heme metabolism proteins are prominent among PPB-bound proteins in fibroblasts, which undergo PPIX-mediated death determined to occur through ferroptosis. PPB proteomic data (available at PRIDE ProteomeXchange # PXD042631) reveal that redox proteins from PRDX family of glutathione peroxidases interact with PPIX. Targeted gene knockdown of the mitochondrial PRDX3, but not PRDX1 or 2, enhance PPIX-induced death in fibroblasts, an effect blocked by the radical-trapping antioxidant, ferrostatin-1. Increased PPIX formation and death was also observed in a T-lymphoblastoid ferrochelatase-deficient leukemia cell line, suggesting that PPIX elevation might serve as a potential strategy for killing certain leukemias.

## Abstract (web summary)

A biotin-conjugated PPIX probe is developed and used to identify PPIX-interacting proteins. One of the identified interacting partners, PRDX3, is found to modulate PPIX-dependent cell death in fibroblasts as well as in a leukemia cell line.

## Introduction

Heme is a prosthetic group for enzymes in key cellular processes such as electron transport, detoxification, protection against oxygen radicals, and oxygen transport^1–5^, and can regulate transcription^4,5^. Heme synthesis requires protoporphyrin IX (PPIX), formation^6^, which is converted into heme by the insertion of ferrous iron by ferrochelatase (FECH)^7–10^. Formation of PPIX and heme are promoted by exogenous addition of the precursor, δ-aminolevulinic acid (ALA)^11,12^. ALA treatment, to elevate PPIX, in combination with light therapy to promote phototoxicity, is the basis of some cancer therapies^13^. PPIX mediated phototoxicity has been well studied, hence, it is known that the mechanism of death, whether by apoptosis, necrosis or some other mechanism, varies between systems^14^. PPIX levels may also become elevated in certain disease states, such as porphyrias^15^, of which many there are several^16,17^. In these diseases, PPIX and other porphyrin precursors accumulate due to genetic deficiencies of the synthetic enzymes of the heme pathway. Similarly, elevated porphyrin concentrations may occur secondarily to environmental toxicants (e.g., lead, arsenic) or other chemicals that disrupt heme biosynthesis in mammals^18^. It is unknown if PPIX-interacting proteins modulate cell death after PPIX elevation, due mostly to our lack of knowledge about these proteins. Comprehensive knowledge of PPIX interacting proteins would be beneficial to developing strategies to enhance cancer therapies, or to ameliorate the toxic side effects of PPIX. While PPIX cytotoxicity can occur without photoactivation, the mechanism is still unclear^19,20^. In some cell types, PPIX interaction with the voltage-dependent anion channel (VDAC) elicits mitochondrial permeability pore opening, a lethal event^21^, in other systems little is known. Knowledge of the differences in protein interactions among tissues with varying sensitivity to PPIX would aid in our understanding.

PPIX protein interactions might also provide insight into its intracellular trafficking. PPIX is formed in the mitochondrial matrix^22^, yet excess may broadly distribute throughout the cell to the plasma membrane, cytosol, mitochondria, endoplasmic reticulum, Golgi, and nuclear envelope^23–30^, suggesting that it is either actively exported or chaperoned from the mitochondria after its synthesis in the mitochondrial matrix. Intracellular PPIX transit likely occurs by interactions with yet-unknown proteins. Some interactions have been documented, such as PPIX binding to the mitochondrial peripheral benzodiazepine receptor^31^, and it is known that PPIX is ultimately exported from the cell by the plasma membrane ABC transporter, ABCG2^32–35^, but the intracellular route to the plasma membrane remains undetermined.

It is unknown as to how many proteins PPIX interacts with, and whether PPIX-interacting proteins differ among cell types. Additionally, it is not known whether the proteins involved in PPIX interactions are conserved among species. Hence, identifying proteins that interact with PPIX might lead to discovery of new biological and functional relationships. Fibroblasts readily form PPIX when provided with the porphyrin precursor, ALA^36^. To identify PPIX-binding proteins we developed a PPIX probe, referred to as PPB, by conjugation to biotin. This PPIX-conjugate interacted with albumin, a known PPIX-binding partner, but was less potent in binding than PPIX^37^. Nonetheless, PPB bound and readily captured proteins in cell lysates This approach was then used to evaluate PPB-interacting proteins in multiple cell lineages, which were then quantitatively determined by a non-labeled approach of tandem mass tag (TMT) proteomics^38^. We identified multiple PPB-binding proteins that are distributed in multiple subcellular compartments which were common across all cell lineages employed but differed quantitatively. Our data clustering analysis of PPB-interacting proteins among four different cell lineages revealed that NIH3T3 fibroblast cells were quantitatively distinct from the other cell lineages. Some PPB-interacting proteins in the fibroblasts were common to the heme biosynthetic pathway. This was noteworthy as, of the four cell lines, only NIH3T3 exhibited cell death due to PPIX formation after exposure to ALA. PPIX toxicity in NIH3T3 was modulated by iron and ROS scavengers, and PPIX induced both cellular and mitochondrial ROS while depleting reduced glutathione, despite increased GPX4 activity. Cell death by PPIX formation was blocked by treatment with either ferrostatin-1 or liproxstatin-1, both powerful inhibitors of ferroptotic cell death^39,40^.

We hypothesized that ROS produced due to PPIX formation might be mitigated by a protein captured by PPB. Comparing our PPB screen results against databases of redox proteins, we identified the antioxidant peroxidases, PRDX1-3, but no other antioxidant enzymes. Genetic suppression of PRDX3, but not the other PRDX members, increased the toxicity of PPIX in NIH3T3. We then extended this hypothesis to determine if cancer cells with a heme synthesis defect might also be susceptible to PPIX mediated death. Finding a T-cell leukemia cell line with FECH deficiency, Jurkat, we demonstrated that these cells were sensitive to the cytotoxic effects of PPIX. The cytotoxicity of elevated PPIX in Jurkat cells was also blocked by ferrostatin-1.

Together our results show that a small molecule probe, PPB, when coupled with proteomic techniques, allowed us to identify conserved interacting proteins from multiple cell types. We were subsequently able to identify one modulator of PPIX-induced death, PRDX3. This approach could similarly be used to identify other currently unknown relationships between biologically active molecules and their metabolic pathways.

## Results

### Increasing PPIX synthesis and cell death

**Fig. 1:**
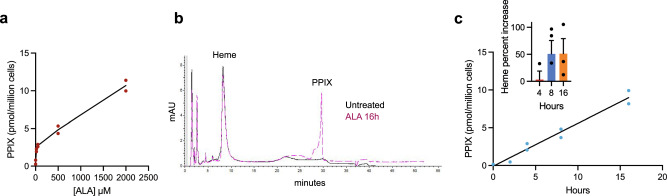
ALA increases PPIX levels dose- and time-dependently without altering levels of other porphyrins. **a** ALA enhances generation of PPIX in NIH3T3 cells in a dose-dependent manner following 6-h treatment with various ALA concentrations. (*n* = 2 biologically independent samples, representative of two independent experiments). **b** Overlay of untreated and 1-h 2 ALA-treated NIH3T3 cells demonstrates marked induction of PPIX but no other changes in heme or other porphyrins. **c** Time dependence of PPIX and heme (insert) generations in NIH3T3 cells treated with 200 µM ALA. (*n* = 2 biologically independent samples, representative of three independent experiments).

**Fig. 2:**
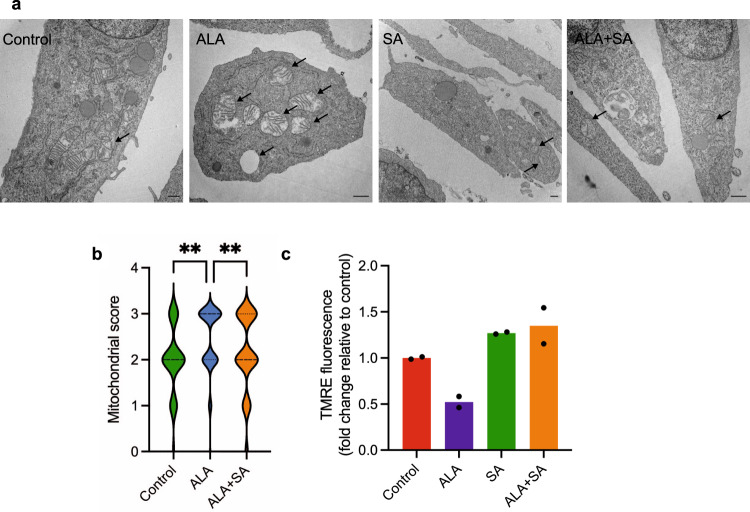
Increased heme/porphyrin synthesis produces altered mitochondria in NIH3T3 cells. **a** Electron micrographs of representative mitochondria used for scoring of morphological abnormalities incurred by ALA treatment (24 h). Arrows indicate mitochondria showing a score of 2–3 in representative slides. Scale bars indicating length are as follows, untreated, succinylacetone and ALA + succinylacetone bars indicate 600 nm, for ALA bar indicates 1 µm. **b** Average scores of mitochondrial morphology change after ALA treatment (illustrated by violin plot where thickness represents distribution); mitochondria viewed by electron microscopy at 6 h were scored with 0 indicating unaltered morphology and 3 indicating severe, dose-dependent impairment (number of mitochondria evaluated were: *n* = 71 for Control, *n* = 88 for ALA and *n* = 108 for ALA + SA). **c** Mitochondrial membrane potential changes with ALA treatment (200 µM). (*n* = 2 independent replicates representative of five independent experiments). Significance is indicated by the number of attached asterisks. *p < 0.05, **p < 0.01, ns indicates no significance. Significance in (**b**) was evaluated using one-way ANOVA, while unpaired Student’s *t* test, two-sided, was used to determine significance in (**c**).

PPIX biosynthesis was increased in NIH3T3 cells by addition of the heme precursor aminolevulinic acid (ALA). ALA produces both dose- and time-dependent increase in heme and PPIX (Fig. 1a, c). Heme was increased only modestly in response to ALA, while no other biosynthetic porphyrin intermediates were detected in the cells (Fig. 1b). As PPIX is generated within the mitochondria and is known to produce mitochondrial damage^41,42^, we investigated whether PPIX affected mitochondrial morphology using transmission electron microscopy (Fig. 2a). Using blinded assessors, we assessed the condition of mitochondria in our treatment conditions. ALA treatment strongly increased the proportion of cells with altered mitochondrial morphology (deformed and/or reduced or lost cristae) (Fig. 2b). These morphological changes were blocked by succinylacetone (SA), an inhibitor of the porphyrin synthetic pathway (Fig. 2a, b). We next investigated whether mitochondrial membrane potential was altered by PPIX elevation using the mitochondrial polarity-sensitive dye, TMRE. Mitochondria showed strong depolarization coinciding with PPIX formation but were not depolarized when PPIX synthesis was blocked with SA, indicating that mitochondrial depolarization requires the formation of porphyrins (Fig. 2c).

### PPIX-biotin (PPB): a probe to capture protoporphyrin IX (PPIX)-binding proteins

**Fig. 3:**
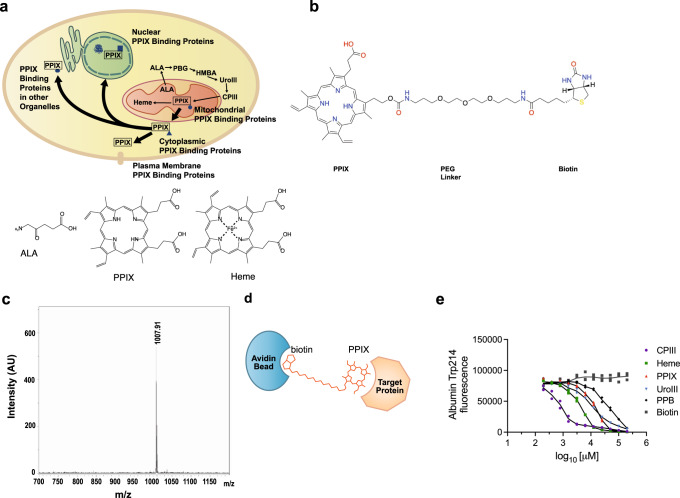
Development and testing of PPB, a PPIX-biotin probe. **a** Model illustrating formation and movement of PPIX within the cell and molecular structures of ALA, PPIX, and heme. **b** Structure of PPB, with PPIX, linker and biotin domains indicated. **c** LC-MS spectrum of synthesized PPB indicating high level of purity. **d** Model indicating the dual interactions of PPB with both PPIX-binding proteins and avidin. **e** PPB binds albumin and quenches fluorescence comparably to PPIX. Fluorescence quenching was in the order, CPIII > Heme > UroIII > PPIX > PPB. (*n* = 2 independent samples, representative of three independent experiments).

Despite the ubiquity of heme synthesis in respiring mammalian cells, there is no comprehensive knowledge of the intracellular proteins that interact with PPIX, its immediate precursor. PPIX synthesis occurs in the mitochondrial matrix, from which it is excluded from mitochondria by an unknown process, and subsequently expelled from cells by the transporter ABCG2 and possibly other transporters (Fig. 3a). To address this gap in knowledge, we developed a probe, PPB, to identify PPIX-binding proteins. We reasoned that a biotin moiety, tethered to PPIX via a linker, might capture PPIX-binding proteins in conjunction with avidin-beads (Fig. 3b). The molecular weight of PPB was confirmed by mass spectrophotometry (Fig. 3c). A diagram of how PPB might interact with a target protein and how the PPB-interacting proteins would be captured by an avidin-bead is shown (Fig. 3d).

### PPIX biotin probe (PPB) protein interaction

**Fig. 4:**
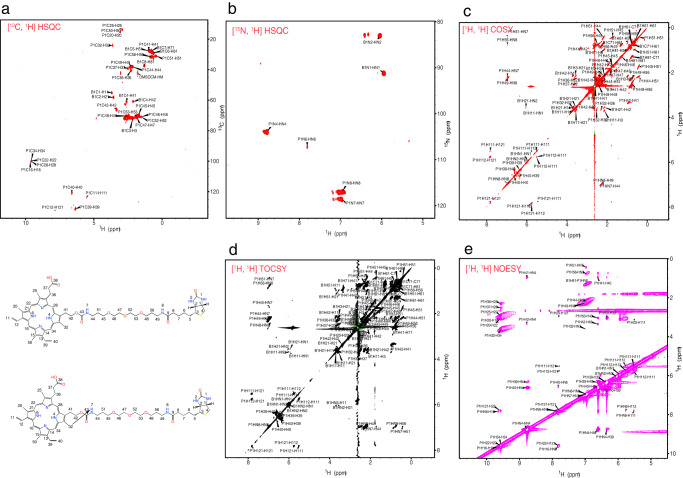
Confirmation of the structure of PPB by NMR. NMR data indicating **a** ^13^C, ^1^H-HSQC, **b** ^15^N, ^1^H-HSQC, **c** ^1^H ^1^H COSY, **d** ^1^H ^1^H TOCS, and **e** ^1^H ^1^H NOESY data confirm the proposed structure of PPB.

We anticipated that our design would allow capture of PPIX-binding proteins by avidin beads (Fig. 3d). We first tested whether the PPIX moiety of PPB was able to interact with proteins comparably to unbound PPIX. We evaluated this by observing whether PPB was able to quench fluorescence in albumin through interaction with tryptophan-214, a known property of other porphyrins, including PPIX^37,43–45^ (Fig. 3e). PPB quenched albumin fluorescence with the slope of the dose-response curve being comparable to PPIX and other porphyrins tested. The slightly lower binding efficiency observed with PPB suggests the added biotin moiety affects its binding; however, biotin alone does not affect albumin fluorescence (Fig. 3e). PPB readily bound to albumin forming a complex, as shown by the passage of PPB through a Sephadex G-25 size-exclusion column in the presence of albumin, whereas free unbound PPB was retained. This indicated that the majority of PPB readily bound albumin (Supplementary Fig. 1a–d). The structure of PPB was confirmed with 2D NMR spectra, and chemical shift assignments of PPB (Fig. 4a–e and Supplementary Table 1 in the Supplementary Information file) show agreement with the proposed structure (Fig. 3b). However, PPIX symmetry does not permit determination as to which position, C13 or C17, the propionate sidechain attaches.

The PPB-binding assessment was extended to cell lysates from U251 cells. To evaluate binding and evaluate potential controls, lysates were incubated with PPB alone and with potential control conditions. These controls included PEG-biotin, which lacks the PPIX moiety, and PPB in addition to excess PPIX or biotin. The color of the pellets was monitored over successive washes as an indicator or porphyrin attachment (Supplementary Fig. 2a). Interestingly, beads incubated with the combination of both PPB and excess PPIX showed a darker purple hue than with either alone, which was not fully removed by washing. This indicated a strong association of PPIX with the PPB bound beads as depicted in the model (Supplementary Fig. 2b), labeled “PPIX competition”. Silver staining and mass spectrophotometry (Supplementary Fig. 2b “PPIX Aggregation”, 2c, d) verified that PPB combined with excess PPIX leads to trapping of proteins and was not further used as a control. Consequently, biotin competition and PEG-biotin controls were used in subsequent experiments.

### Identification of PPB-interacting proteins

**Fig. 5:**
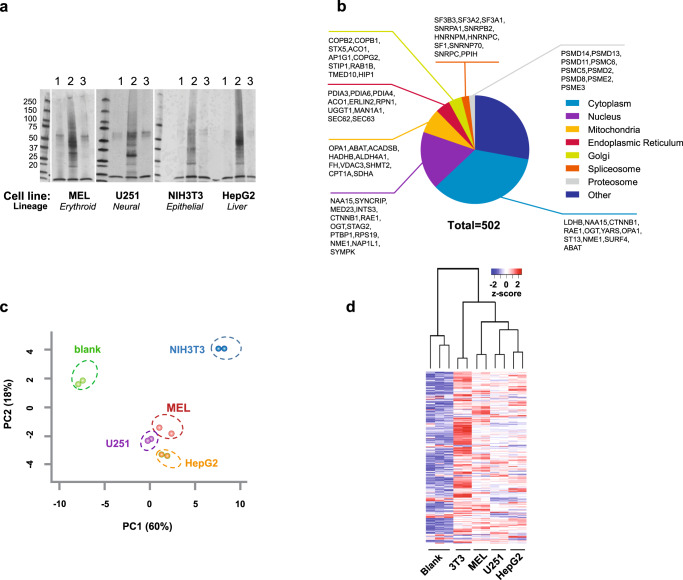
Mass spectrometry using tandem mass tag labeling indicates cellular differences in PPIX-binding proteins. **a** Representative silver stain of PPB immunoprecipitation and controls from four cell lines (*n* = 3 or more independent experiments for each cell line). **b** Subcellular localization of PPIX-binding proteins identified by TMT. **c** Principal component analysis (PCA) of TMT proteomics data. **d** Unsupervised hierarchical clustering of cell lines’ quantitative TMT proteomic data from PPB immunoprecipitation assay.

To identify PPB-interacting proteins, PPB-bound proteins were precipitated from cell extracts from four distinct cell lines (Fig. 5a). These cell lines, taken from four different lineages, included NIH3T3, a mouse fibroblast cell line; MEL, a mouse erythroblast cell line; HepG2, a human liver cell line; and U251, a human glioblastoma cell. Cell lines were chosen for their diversity with respect to physiological function and heme/porphyrin needs. PPB-interacting proteins were identified and quantitated for each species and lineage for our four cell lines by quantitative mass spectrometry (MS) using tandem mass tag (TMT) analysis^46^. For each cell line, three independent cell lysates were prepared and incubated with PPB, or controls. In total, 543 discrete specific candidates were identified as PPB-interacting proteins (FDR < 0.01 and *z*-score >3; Supplementary Data 1, see tab Data Candidates). Multiple pathway analysis programs and gene ontology databases (KEGG, GO, and Hallmark, etc.) were interrogated to identify pathways common to these PPB-binding proteins, revealing pathways relevant to mitochondria, heme/porphyrin synthesis, transport, necroptosis, and metabolism (Supplementary Data 2, see tabs Enrichment function, Enrichment process, Keyword, Pfam, KEGG, component and RCTM (reactome)). These PPB-interacting proteins localized to multiple subcellular compartments, with the largest portion (35%) being cytosolic (Fig. 5b). Among the PPB-bound proteins identified was a recently reported PPIX-binding protein, elongation factor alpha, EEF1A1^20^ (Supplementary Data 1). Using immunoblotting we were able to validate several binding candidates, including ANT1 and ANT2 (Supplementary Fig. 3a), consistent with their proposed role as PPIX transporters^47^. Likewise, two proteins, SDHA and SDHB, which are part of a complex, were independently identified by immunoblot (Supplementary Fig. 3a). Importantly, SDHC, which is physically distinct from SDHA and B, was not pulled down.

**Fig. 6:**
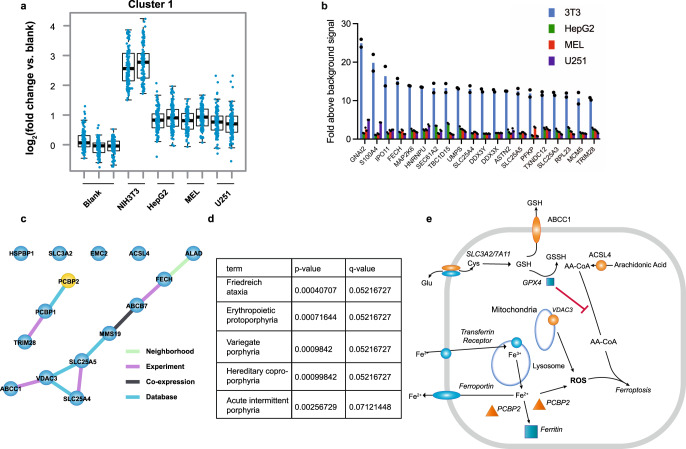
Analysis of PPIX-interacting proteins identifies NIH3T3-specific cluster of PPIX-binding proteins related to iron and mitochondrial metabolism. **a** Weighted gene co-expression network analysis (WGCNA) of the PPB and tandem mass tag (TMT) quantitative proteomics data identified a cluster of proteins enriched in NIH3T3 cells. **b** Highest candidate PPIX-binding proteins from NIH-3T3 cluster 1 shown relative to other cell lines (*n* = 2 biologically independent samples, mean). **c** STRING protein-protein interaction (PPI) network analysis of cluster 1 identifies a protein module enriched for iron and oxidative phosphorylation. **d** Enrichr analysis of cluster 1 identifies porphyria and iron relationships. **e** PPIX-binding proteins identified in screen (orange) demonstrating a role in iron pathway and ferroptosis.

Seeking to learn more about the differences between PPIX binding in the cell lines examined, we employed principal component analysis and unsupervised clustering of PPB-bound proteins, which revealed that Mel, HepG2, and U251 cells segregated together (Fig. 5c), while NIH3T3 cells were distinct (Fig. 5c, d). An unweighted gene-clustering analysis^48^ revealed eight distinct clusters among the PPB-binding proteins^49^ (Supplementary Fig. 4 and Supplementary Data 3). Among these clusters, many proteins were in common among the various cell lines, however, one cluster (cluster 1) segregated the NIH3T3 cells from the other cell lines (Fig. 6a).

Because of the relationship between PPIX formation and toxicity in NIH3T3 cells we further analyzed the NIH3T3-specific cluster. Highly enriched PPB-interacting proteins in the NIH3T3-specific cluster included many that were strongly related to heme and iron metabolism (Fig. 6b). This was confirmed by additional analyses with String, Cytoscape and Enricher, which shows the interdependency between pathways (Fig. 6c, d). We consider it likely that connections among these PPB-binding proteins might be further explored to uncover relationships between PPIX biosynthesis, cell death and iron metabolism. Interestingly, some PPB proteins were also linked to the ferroptosis pathway (Fig. 6e).

**Fig. 7:**
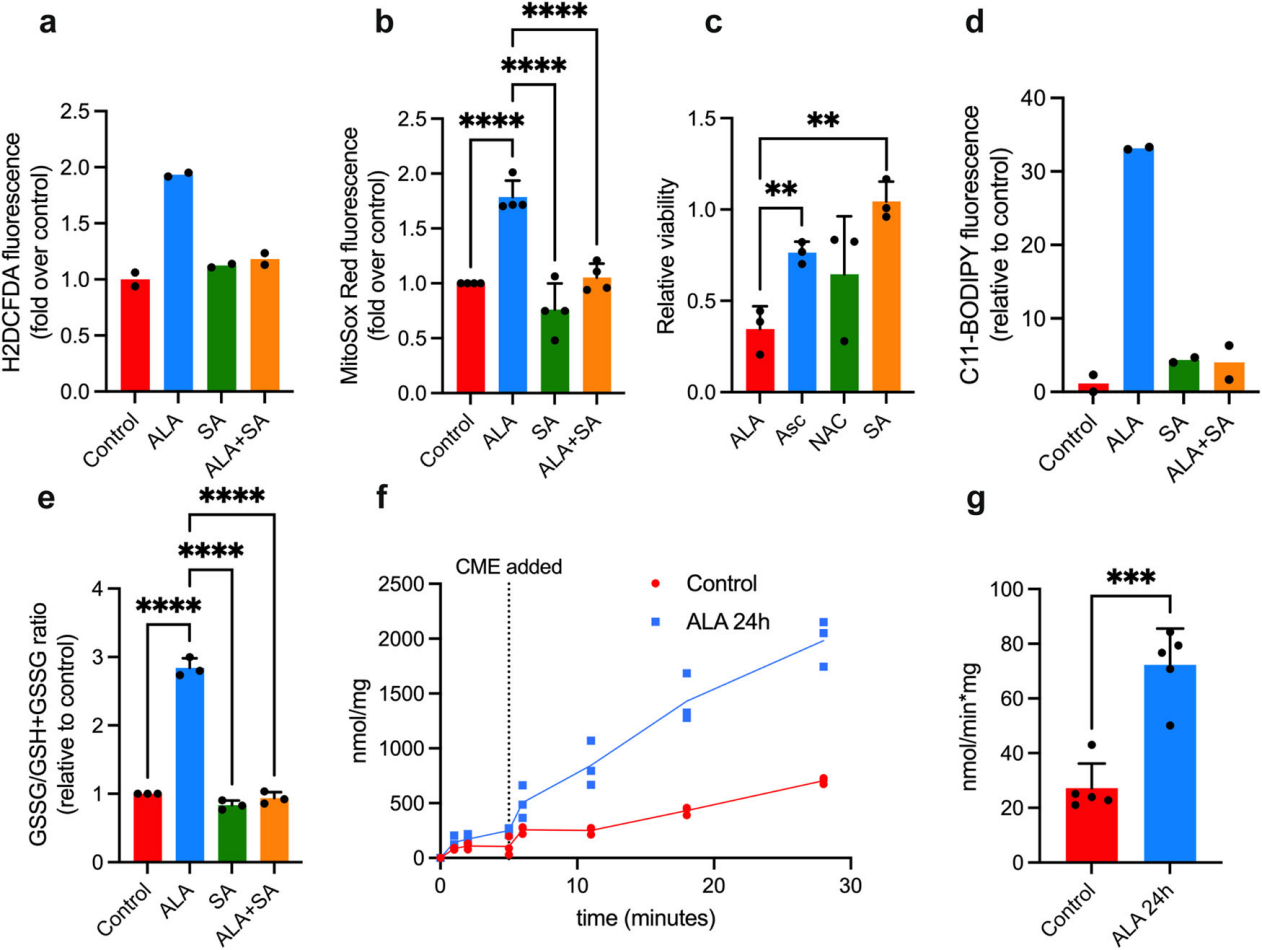
ALA induces increases ROS and promotes ferroptosis. **a** Total cellular ROS in ALA-treated cells, as measured by DCFDA fluorescence (*n* = 2 independent samples, representative of three independent experiments). **b** Mitochondrial superoxide levels in ALA-treated cells, as measured by MitoSOX Red fluorescence (*n* = 4 independent experiments, mean ± SEM). **c** ALA cytotoxicity is suppressed by antioxidants; ascorbic acid (500 µM), N-acetylcysteine (5 mM) or ferrostatin-1 (10 µM) (*n* = 3 independent samples, representative of 3 or more independent experiments for all drugs tested, mean ± SEM). **d** Increased lipid peroxidation with ALA treatment and attenuation by SA, as assessed by C11-Bodipy (*n* = 2 independent samples, representative of three independent experiments). **e** Oxidized/total glutathione ratio changes in ALA-treated cells or cotreated with succinylacetone (*n* = 3 independent experiments, mean ± SEM). **f** Glutathione peroxidase activity of lysate from NIH3T3 in control or 24-h ALA (200 µM) treated, following addition of cumene hydroperoxide substrate (*n* = 3 independent samples, representative of five independent experiments, mean ± SEM). **g** Specific activity of glutathione peroxidase in control and 24-h ALA-treated NIH3T3. Significance is indicated by the number of attached asterisks (*n* = 5 independent samples, representative of five independent experiments, mean ± SEM). *p < 0.05, **p < 0.01, ***p < 0.001, ****p < 0.0001, and ns indicates no significance. Significance in this figure was evaluated using one-way ANOVA.

NIH3T3 cells with elevated PPIX levels, following ALA treatment, displayed increased ROS as measured with dichlorodihydrofluorescein diacetate (H2DCFDA) (Fig. 7a). MitoSOX Red showed that ALA treatment also increased mitochondrial superoxide ROS (Fig. 7b). Each ROS increase required the formation of PPIX, as SA blocked the increase in ROS and PPIX. Viability was also strongly reduced by ALA treatment but rescued by antioxidant supplementation with ascorbate (ASC) and N-acetylcysteine (NAC), or by inhibiting porphyrin synthesis with succinylacetone (SA) (Fig. 7c). Erastin, an inducer of ferroptosis which does not increase porphyrin levels, was not affected by SA treatment, as expected (Supplementary Fig. 5).

ALA treatment increased C11-BODIPY peroxidation, a fluorescent lipid peroxidation reporter (Fig. 7d). The antioxidant enzyme GPX4, a glutathione peroxidase, protects against peroxide damage by using glutathione to defend the cell against ROS. Depletion of glutathione paralleled the increase in PPIX and was accompanied by increased formation of oxidized glutathione (Fig. 7e). This indicates that sustained PPIX formation creates an oxidative challenge to the cell by depleting reduced glutathione, necessary for cellular protection against lipid hydroperoxides by the enzyme GPX4. We next determined whether GPX4 activity was altered in cells with activated PPIX synthesis. We hypothesized that PPIX might diminish GPX4 activity, interestingly, however we found it was increased by over two-fold (Fig. 7f, g), indicating that it was not a key element in PPIX induced toxicity.

**Fig. 8:**
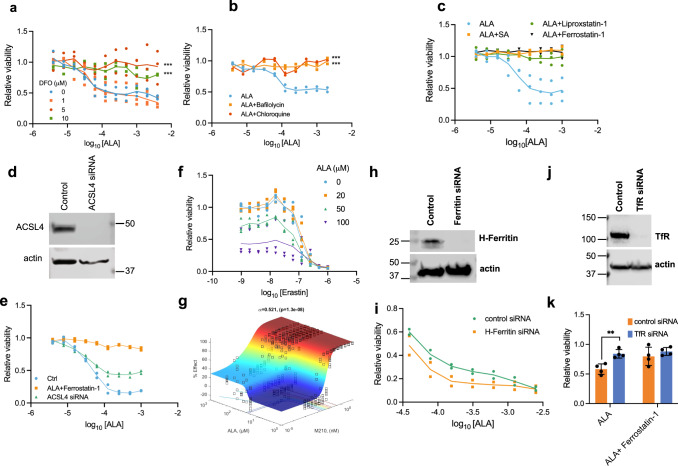
Modulators of iron, endocytosis, and lysosome fusion alter ALA toxicity. **a** Dose-dependent reduction of ALA cytotoxicity by desferrioxamine (DFO) iron chelation treatment (*n* = 3 independent experiments). **b** ALA cytotoxicity is reversed by both chloroquine (50 µM) and bafilomycin (20 nM) (*n* = 2 independent samples, representative of three independent experiments). **c** ALA cytotoxicity is reversed by ferrostatin-1 (10 µM) and liproxstatin-1 (10 µM) (*n* = 3 independent experiments). **d** Knockdown of ACSL4 in NIH3T3 or control siRNA at 48 h (*n* = 2 independent experiments). **e** Rescue of ALA toxicity of NIH3T3 by ferrostatin-1 (10 µM) or by ACSL4 knockdown 48 h prior to treatment. (*n* = 2 independent samples, representative of two independent experiments). **f** Co-treatment of cells with ALA and various concentrations of erastin increased cytotoxicity (*n* = 2 independent experiments). **g** Response surface modeling analysis of ML210 and ALA cotreatment in NIH3T3, *α* = −0.521, analysis of datasets from four independent experiments. **h** Immunoblot analysis of H-ferritin expression treated with either control siRNA or specific H-ferritin siRNA. (*n* = 2 biologically independent experiments). **i** Gene knockdown of H-Ferritin sensitizes cells to ALA cytotoxicity (*n* = 2 biologically independent experiments). **j** Immunoblot analysis of TfR expression treated with either control siRNA or specific TfR siRNA. cytotoxicity (*n* = 4 independent, mean ± SEM). **k** Gene knockdown of TfR protects cells from ALA cytotoxicity (*n* = 4 independent experiments, mean ± SEM). *p < 0.05, **p < 0.01, ***p < 0.001, ****p < 0.0001, and ns indicates no significance.

To determine if chelatable iron contributes to ALA-induced PPIX synthesis cytotoxicity, cells were cotreated with the iron-chelator, desferrioxamine (DFO), which dose-dependently suppressed ALA-induced cell death (Fig. 8a). Cell cycle was not altered by increased PPIX formation (Supplementary Fig. 6). To better understand the role of iron in PPIX-promoted cell death, we investigated iron uptake. Iron can be chaperoned into the cell by the transferrin receptor (TfR1) or by H-ferritin^50,51^ and subsequently released after fusing with the lysosome^31^. Iron is also sequestered in a complex formed by H-ferritin and L-ferritin^52^. We reasoned those inhibitors of lysosome function, chloroquine and bafilomycin, might restrict lysosomal iron uptake. Both agents attenuated PPIX-induced cell death (Fig. 8b). The role of clathrin-mediated endocytosis (CME) was assessed using the dynamin inhibitor, Dynasore (DS), or one of two other CME inhibitors, sucrose and Pitstop2, as off-target effects have been demonstrated with Dynasore^53–55^. Each inhibitor enhanced viability under ALA treatment (Supplementary Fig. 7a) demonstrating that endocytosis is important. Only Dynasore rescued erastin toxicity (Supplementary Fig. 7b). While ALA and sucrose treatment each produced increases in membrane associated H-ferritin, the combination resulted in a larger increase than either alone, suggesting that ALA treatment drives changes in endocytic iron uptake (Supplementary Fig. 7c).

Given the involvement of iron and ROS, we investigated whether ferrostatin-1 and liproxstatin-1, antioxidants which prevents lipid peroxidation and ferroptosis, might block PPIX toxicity. Intriguingly, both blocked cell death (Fig. 8c). Next, we tested whether siRNA knockdown of ACSL4 acyl-CoA synthetase, a PPIX-binding protein implicated as a driver of ferroptosis (Supplementary Data 1), might affect ALA toxicity. Partial siRNA knockdown (Fig. 8d) provided protection against ALA-induced death (Fig. 8e), further implicating ferroptosis.

We next performed experiments to examine the effect of cotreatment of NIH3T3 with ALA and known inducers of ferroptosis. Treatment with ALA and erastin individually and in combination produced dose-dependent toxicity (Fig. 8f). Surprisingly, analysis of the interaction showed it to be antagonistic (Supplementary Fig. 8). Similar analysis, however, with ferroptosis inducer, ML210, which acts through inhibition of GPX4, demonstrated a synergistic relationship (Fig. 8g), implying that the mechanisms reinforce each other. We performed knockdown experiments to address a role for ferritin, which plays roles in both iron transport and storage in ALA toxicity. Using a H-ferritin siRNA knockdown (Fig. 8h) we found PPIX-cytotoxicity was enhanced by H-ferritin knockdown over a broad range of ALA concentrations (Fig. 8i). We then interrogated the role of TfR in death mediated by PPIX. Iron-bound transferrin binds the transferrin receptor, TfR, followed by internalization and endosome formation^56^. Knockdown of TfR (Fig. 8j) reduced the PPIX-induced cytotoxicity (Fig. 8k). Overall, we interpret these findings to indicate that both H-ferritin and the TfR independently play important roles in PPIX- induced cell death consistent with ferroptosis.

### Redox protein interactions with PPB, including PRDX3 that modulates PPIX death

**Fig. 9:**
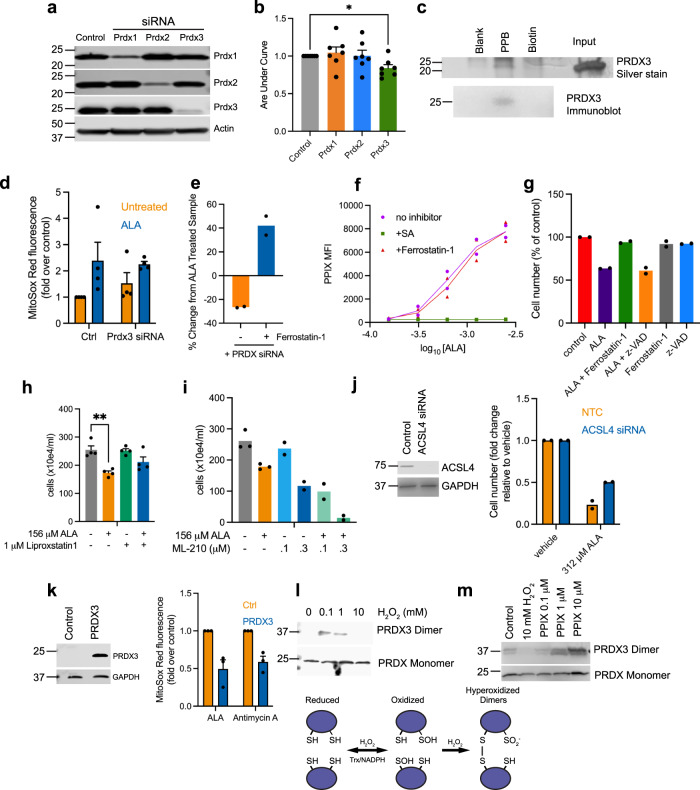
ALA mediated cell death is distinct from erastin mediated ferroptosis, is enhanced specifically by knockdown of PRDX3, and increases PPIX and toxicity in Jurkat. **a** Efficient knockdown of PRDX1, 2, and 3 by siRNA treatment (*n* = 7 experiments). **b** Area-under-the-curve assessment of graphs of ALA-treated cells, with-or-without suppression of PRDX1, 2 or 3. Knockdown of PRDX3, but not that of PRDX1 or PRDX2, enhances the cell death caused by PPIX induction (*n* = 7 independent experiments, mean ± SEM). **c** Pulldown of PRDX3 from NIH3T3 lysate and silver staining (top) or immunoblot for PRDX3 (bottom). (*n* = 2 independent experiments). **d** MitoSOX Red mitochondrial superoxide indicator fluorescence increases in NIH3T3 with ALA treatment in both PRDX3 knockdown and control (*n* = 4 independent experiments, mean ± SEM). **e** ALA (156 μM) induced cytotoxicity is potentiated by siRNA PRDX3 knockdown, but reversed by ferrostatin-1, but which was reversed by the addition of ferrostatin-1 (*n* = 2 independent experiments). **f** ALA dose-dependently increases PPIX fluorescence in Jurkat cells (*n* = 2 independent experiments). **g** ALA reduced Jurkat cell viability is attenuated by ferrostatin-1 (10 µM), but not ZVAD (20 µM). (*n* = 2 independent experiments). **h** Jurkat cell viability is rescued by liproxstatin-1 under ALA treatment, 48 h. (*n* = 4 independent experiments, mean ± SEM). **i** ML210 and ALA produce additive effect on Jurkat viability. (*n* = 3 independent experiments, mean ± SEM). **j** ACSL siRNA knockdown rescues ALA toxicity in Jurkat cells. (*n* = 2 independent experiments). **k** PRDX3 overexpression in 293T cells reduces MitoSOX Red fluorescence induced by ALA or antimycin A. (*n* = 3 independent experiments, mean ± SEM). **l** PRDX3 from NIH3T3 treated with hydrogen peroxide (5 min) shows increased levels of hyperoxidized dimeric form shown in model. **m** PPIX treatment of NIH3T3 induces formation of hyperoxidized dimers. Total PRDX3 is shown following reduction with β-mercaptoethanol. (*n* = 2 biologically independent experiments) *p < 0.05, **p < 0.01, ***p < 0.001, ****p < 0.0001, and ns indicates no significance.

As PPIX formation elevated ROS, while antioxidants proved protective against toxicity, we queried available databases for potential antioxidant enzymes among PPB-binding candidates, finding few. We noted, however, that three of the six peroxiredoxin family members, PRDX1-3, bound PPB. PRDX proteins are ubiquitous cysteine-dependent antioxidant enzymes that metabolize peroxides^57^. Suppression of PRDX 1-3 with siRNAs revealed that selective suppression of each respective PRDX family member was possible (Fig. 9a). Cell viability as a function of ALA concentration was assessed in cells suppressed in one of the three PRDX family members as well as control cells. When the area-under-the-curve (AUC) for each graph was calculated and plotted as a measure of toxicity across the full dose range of ALA, toxicity was found to increase when PRDX3, but not PRDX1 or PRDX2, was suppressed (Fig. 9b). Confirmation of PRDX3 binding to the PPB probe (Fig. 9c) suggested a mechanism in which direct binding of mitochondrially produced PPIX might suppress ROS quenching by PRDX3, increasing mitochondrial superoxide. ALA treatment increased the levels of mitochondrial ROS in both control and PRDX3 knockdown cells (Fig. 9d). Further, PRDX3 knockdown impacted PPIX-mediated death, as cells treated with PRDX3 siRNA and ALA had a greater viability loss (27%) relative to treatment with ALA alone (Fig. 9e).

### PPIX biosynthesis, and cell death in a T-cell lymphoblastic leukemia

Because of the relationship between PPIX formation and cell death in cancer cells^12,58^, we interrogated the cancer dependency map (DepMap) for cell lines reliant on the terminal enzyme in heme biosynthesis, FECH. Among them we discovered the T-lymphoblastoid cancer cell line, Jurkat, with a *FECH* frame shift deletion, accounting for its very low ferrochelatase activity^59^. Next, we investigated whether ALA treatment produced elevated intracellular PPIX in Jurkat cells. Using FACS analysis, we found that ALA dose-dependently increased intracellular PPIX (Fig. 9f). We then evaluated whether elevated PPIX promoted cell death in Jurkat cells, and whether that death occurred by a process inhibitable by ferrostatin-1. Cells were treated with either ALA, ALA and ferrostatin-1, ferrostatin-1 alone, or ALA and ZVAD-FMK, the pan-caspase inhibitor (Fig. 9g). Cell viability was reduced by almost 50% with ALA treatment and viability was not restored by the addition of ZVAD-FMK. Only, ferrostatin-1 was capable of rescuing Jurkat cells from death produced by elevated intracellular PPIX. Further testing with liproxstatin-1 substantiated this finding (Fig. 9h), while treatment with the GPX4 inhibitor, ML210, which promotes ferroptosis, led to a synergistic toxicity with ALA treatment (Fig. 9i). Furthermore, knockdown of the ACSL4 long-chain fatty acid synthase improved Jurkat survival under ALA treatment (Fig. 9j), consistent with ferroptosis. These findings suggest that the ferroptotic potential for PPIX induction extend beyond NIH3T3, and that certain cancer cells might be killed through increasing PPIX levels via ferroptosis.

As our findings in NIH3T3 and Jurkat demonstrated that reduction of PRDX3 modulates ROS and ALA toxicity we investigated the effect of overexpression. Overexpression of PRDX3 in 293T cells powerfully reduced the excess superoxide ROS generated by both ALA and the control, antimycin A (Fig. 9k). We then attempted to determine whether PPIX might directly reduce PRDX3 activity. The direct effect of PPIX on PRDX3 activity proved technically challenging to assess. It is known, however, that PRDX may be converted to an inactive overoxidized dimeric form, as occurs with exposure to high concentrations of hydrogen peroxide^60,61^ (Fig. 9l). PPIX dose-dependently induced a similar shift to the formation of this dimer (Fig. 9m), implying that it may interact and produce inhibition.

## Discussion

Previously, probes (such as hemin) coupled to agarose beads have been employed to inventory the heme-binding proteins in cells or tissues^62^. However, PPIX, a prominent tetrapyrrole intermediate in the heme biosynthetic pathway, has never been used to probe for potential PPIX protein interactions^63^ to the best of our knowledge. Here, we describe the development, characterization, and use of a PPIX probe (PPB). Coupled with proteomics, PPB binding broadly interrogates the PPIX interactome. While cellular levels of PPIX relative to heme in normal cells are ordinarily low^64^, the relatively slow rate of heme formation by ferrochelatase in many cells, particularly tumors, allows an opportunity for vastly higher ratios when exposed to exogenous ALA^10^. The small degree of heme induction in ALA-treated NIH3T3, relative to PPIX, might suggest limiting ferrochelatase activity, however our TMT data suggest robust ferrochelatase levels. We believe it more likely, therefore, that the iron importation process may limit the production of heme in NIH3T3, leading to PPIX buildup. We initiated these studies because fibroblast cells exhibited a PPIX-dependent cell death that was not related to PPIX phototoxicity and the non-phototoxic mechanisms of PPIX cytotoxicity are unclear. We hypothesized that differences in PPIX binding capacity among cell types might account for this difference in toxicity. To assess PPB binding, lysates from four cell lines, NIH3T3, HepG2, MEL and U251 were used. These represent four different histotypes (two from each, mouse and human) with different capacities to synthesize PPIX/heme. HepG2 hepatoblastoma cells express abundant heme-requiring P450 enzymes^65^, Murine erythroleukemia (MEL) cells readily form heme to accommodate the large amounts of hemoglobin synthesized upon differentiation, and ALA-treated U251 glioblastoma cells accumulate high levels of PPIX^66,67^. We discovered an array of proteins that were common among these cells, but quantitatively different. Further, the PPB-binding proteins distributed throughout multiple subcellular compartments, paralleling PPIX’s broad intracellular immunofluorescence and suggesting this distribution might be due to PPIX-binding proteins^23–30,68–70^. Through curation and extensive pathway analysis (Keyword enrichment, Pfam, KEGG, reactome, GO) of the PPB proteins, we discovered an unanticipated relationship between PPIX formation and cell death. The concurrence of ROS and PPIX led us to investigate whether any of our PPB-binding proteins were antioxidants that might affect PPIX death and ROS. PRDX3, a peroxidase and mitochondrial ROS scavenger^71^, is the only PRDX member that both bound PPB and enhanced the PPIX-driven cell death after siRNA knockdown.

Our approach for capturing cellular PPB-binding proteins proved successful in identifying previously reported PPIX-binding proteins, such as EEF2A1^26^ and ferrochelatase^72^. A literature search revealed that some of our PPB-bound proteins have been reported to bind heme (Supplementary Fig. 9). It is unlikely, however, that heme was formed from PPB in vitro in our assays. Catalytic conversion of the PPIX of PPB to heme via ferrochelatase would require insertion of ferrous iron which would require near anaerobic conditions^73^. Further, little free iron was present. It is more likely that some heme- and PPIX-binding proteins recognize common elements of the tetrapyrrole structure. It is also possible that PPB might be displaced by other intracellular porphyrins (e.g., coproporphyrin III or uroporphyrin III) of greater affinity, however the amounts these free porphyrins were below detection in our cell lysates. Interestingly, ABCG2, a plasma membrane transporter known to bind and export PPIX from the cell, was not detected. The inability to detect PPB interaction with ABCG2 in cell lysates could be due to either the extraction conditions or, perhaps the level of ABCG2 in these cells was too low to be detected.

We noted through PCA analysis of our TMT candidates, an unbiased technique for visualizing differences in datasets, that NIH3T3 cells were distinguishable from the other three cell lines. Cluster analysis revealed a well-defined set of proteins that was more abundant in the NIH3T3 cells. Many PPB-associated proteins were related to iron and heme synthesis, including MMS19^74^, FECH^9^, ABCB7^75^, and both SLC25A4 (ANT1) and SLC25A5 (ANT2), which form the mitochondrial ADP/ATP translocase^76^ and have been shown to import PPIX and heme^47^.

Our analysis of PPIX-binding proteins pointed out differences in NIH3T3 and other cells with respect to PPIX and offered insight to uncover the selective sensitivity of NIH3T3 cells to ALA and to conclude that it occurs through ferroptosis. ALA treatment of NIH3T3 cells readily promoted PPIX generation, but not other porphyrin intermediates in heme synthesis. Heme was only marginally elevated by ALA treatment, likely due to its synthesis being limited by other steps, such mitochondrial iron delivery to ferrochelatase. PPIX induction by ALA in this cell line revealed a susceptibility to PPIX-induced cell death that was not evident in either HepG2 cells or U251 cells. Interestingly, PPIX susceptibility to cell death also differed with respect to the fetal calf serum used to culture the NIH3T3 cells, possibly due to variation of some unknown serum component. Importantly, chemical inhibitors of multiple forms of cell death, such as apoptosis, pyroptosis, or necroptosis (Supplementary Fig. 10) were incapable of preventing PPIX-induced cell death. This is intriguing, as it seems to distinguish the death observed by PPIX induced phototoxicity, which is mediated by a combination apoptosis and necrosis^14,77^. Cell death was, however, readily blocked by succinylacetone, which blocks PPIX formation at ALA-dehydratase, whose substrate is ALA. Significantly, PPIX mediated cell death was readily reversed by both ferrostatin-1 and liproxstatin-1, known ferroptosis inhibitors that block lipid peroxidation, as well as by prototypical ROS scavengers such as N-acetylcysteine and suggesting ferroptosis as the mechanism. While ferrostatin-1 and liproxsatin-1 were not able to fully rescue after PRDX3 knockdown there are possible explanations. It is possible that other forms of death may contribute to toxicity under these conditions, Regardless, most toxicity is allayed by ferrostatin-1 under all conditions tested. Our results indicate that PPIX-induced loss of viability is preceded by increased ROS levels, glutathione depletion, lipid peroxidation and loss of mitochondrial membrane potential. Together, they support the conclusion that PPIX produces ferroptosis in a fibroblast cell line, NIH3T3, a link that we believe to be previously unidentified.

Iron uptake and trafficking contribute to homeostasis of intracellular iron, key element of ferroptosis, and equally important in PPIX-induced ferroptosis. Exogenous iron enhanced ALA-induced cytotoxicity, while chelation with DFO reduced cell death, supporting the role of free exchangeable intracellular iron in PPIX induced cytotoxicity. The lysosomal inhibitors bafilomycin and chloroquine also blocked PPIX induced cell death, implying that intracellular iron trafficking through the lysosome was a key component of PPIX induced cell death. Treatment with inhibitors of endocytosis such as the dynamin inhibitor, Dynasore, as well as sucrose and Pitstop2, blocked PPIX induced cell death. Dynasore blocks death through diverse pathways, not only by inhibiting transferrin receptor endocytosis, but also by mitigating ROS that affects mitochondrial respiration^55^. Consequently, we used other effectors of endocytosis to confirm specificity. Inhibition of endocytosis with hypertonic sucrose also blocked PPIX death, supporting the view that iron transit into the cell by an endocytic process is an important mediator. Iron is bound and then trafficked to the lysozome by plasma membrane receptors such as TfR1 and H-ferritin^78,79^. Elevated PPIX appears to increase H-ferritin levels at the plasma membrane, with greater H-ferritin membrane accumulation when endocytosis (retrieval of H-ferritin) is blocked. Knockdown of H-ferritin enhanced cell death indicating H-ferritin has an overall protective role. We have also shown that siRNA-mediated knockdown of TfR1 blocked PPIX induces death. Collectively, these studies demonstrate that both H-ferritin and TfR1 have distinct roles in modulating the death induced by increased PPIX.

Our data confirmed that ROS is central to PPIX-induced ferroptosis, leading us to consider that antioxidant enzymes might be responsible for some of the toxicity observed and sought to characterize the intersection of PPIX-binding proteins and enzymes affecting redox state (Supplementary Data 4). One candidate was succinate dehydrogenase complex. Based on our discovery that SDHA and SDHB bind to PPIX, we suspected initially that mitochondrial PPIX may inhibit this complex, uncoupling respiration and the TCA cycle, with possible ROS consequences. However, direct treatment with PPIX demonstrated that this enzyme was not strongly affected by PPIX in the lower micromolar range likely to be encountered (Supplementary Fig. 3b). Curiously, ABCC1, which was identified in our screen, can export GSH from the cell, potentially reducing the available supply for proteins such as Gpx4. We considered that PPIX might lead to a gain-of-function for ABCC1 resulting in diminished GSH and producing ferroptotic death. The involvement of ABCC1 was ruled out, however, as inhibition of the transporter provided no rescue (Supplementary Fig. 11), Intriguingly, though, the list included three members of the PRDX antioxidant family, the peroxiredoxins. PRDX are among a class of proteins that scavenge ROS, three of which bound PPB. We hypothesized that suppression of these enzymes, either alone or in combination, might enhance PPIX induced cell death. Only PRDX3 silencing enhanced PPIX toxicity. As PRDX3 localizes to the mitochondria^80^; this result implied that PRDX3 expression might modulate PPIX initiated cell death by blocking peroxidation, similar to how cytosolic PRDX1 has been shown to block ferroptosis in corneal epithelial cells^81^. Coupled with our findings, this suggests that the PRDXs might function to modulate ROS in a subcellular compartment-dependent fashion. Interestingly, we found that ALA-induced ferroptotic cell death was synergistic with canonical ferroptosis inducer ML210, although not with erastin. The difference is not clear, but it may involve the difference in mechanisms between the two agents. ML210, suppresses GPX4 function, while erastin has pleiotropic roles at the mitochondria, with p53 and system XC^−^^82^.

Briefly summarized, our study used a novel PPIX-biotin (PPB) probe to capture PPIX-interacting proteins in four cell lines, diverse with respect to histotype, species, and capacity to form PPIX. We were able to analyze that data and make predictions about PPIX function, resulting in several key findings. First, despite the differential capacity to form PPIX, the proteins captured by PPB were almost identical among cell types but differing in quantity. Second, through gene set enrichment and pathway analysis, we discovered that formation of PPIX promotes ROS formation leads to previously undescribed ferroptotic cell death in NIH3T3 cells. Several of the highly significant PPB-interacting proteins we identified have roles in metabolism-related pathways such as oxidative phosphorylation, ROS, and mTORC1 (see Supplementary Data 5), suggesting that these PPB-biding proteins might modulate these pathways, indicating areas for future studies. Thirdly, we predicted that a ferrochelatase-deficient cancer cell line, Jurkat, might be similarly susceptible to PPIX induced death. This implies that other cancer cells, such as those harboring impaired FECH function, might also be vulnerable to this form of cell death. Finally, we were able to use our data to identify an antioxidant enzyme from our screen, PRDX3, that can modulate PPIX induced cell death. While PRDX3 is only one of undoubtedly many proteins involved in porphyrin-mediated ferroptosis, the techniques we developed might be applied to other small molecules with incomplete interactomes to make connections otherwise not possible.

## Methods

### Compounds

The following reagents were used: d-aminolevulinic acid, bafilomycin, chloroquine, ferrostatin-1, (Sigma), Laemmli buffer (GeneTex), Bodipy 581/591 C11, Phen Green SK, (Invitrogen), TMRE (Abcam), protoporphyrin IX (Frontier), GSK872, necrosulfonamide, (Cayman), Liproxstatin (Tocris), and Dynasore (Abcam). Antibodies used are listed in Supplementary Table 1 of the Supplementary Information File.

### Cell lines

NIH3T3 and U251 cells were cultured in Dulbecco’s modified Eagle’s medium (Gibco) without nucleosides and supplemented with 10% fetal bovine serum (Hyclone for NIH3T3 cells, Gibco for others), 100 units/ml penicillin/streptomycin and 2 mM L-glutamine. MEL cells were cultured in RPIM 1640 medium (Gibco) without nucleosides and supplemented with 10% fetal bovine serum, 100 units/ml penicillin and 2 mM L-glutamine. HepG2 cells were cultured in Minimum Essential Medium α (Gibco) with 10% fetal bovine serum and 100 units/ml penicillin. NIH3T3, HepG2 and Jurkat cells were obtained from ATCC. MEL cells were originally a gift from the lab of Paul Ney while at St. Jude Children’s Hospital. U251 cells were obtained from Sigma.

### Statistics and reproducibility

Significance levels are indicated in legends of each figure. Tests used to derive significance are given. Individual data points are indicated in graphs, along with information as to whether the data represents the composite of multiple biologically independent experiments or replicates of a single experiment representative of multiple experiments. In the latter case all replicates are independent and not multiple measurements taken from a single sample. Analysis of significances was done using one-way ANOVA or, alternately, unpaired Student’s *t* test, two-sided. The tests employed are described in the figure legends. Uncropped and unedited images from all gels are available as supplementary figures in the Supplementary Information file.

### Albumin fluorescence quenching

Ability of PPB to interact with protein was assessed through quenching of albumin fluorescence. Photo-oxidation of either PPB or the other porphyrins was prevented by conducting all manipulations in the dark. The interaction of albumin tryptophan 214 with PPIX and other proteins, causes a loss of intrinsic fluorescence. Bovine serum albumin (BSA) fluorescence was determined by using 4 µM albumin in phosphate-buffered saline (PBS). Each compound was prepared in DMSO and then added to 100 µl of 4 mM albumin in PBS in triplicate lanes of a 96-well plate and allowed to bind for 15 min. Equivalent volumes of DMSO alone were added to negative control wells. Fluorescence was then assessed at wavelengths of 290-nm excitation and 337-nm emission and normalized to untreated albumin. Multiple porphyrins (hemin, PPIX, uroporphyrin III, coproporphyrin III) as well as PPB were tested and compared. Photo-oxidation of either PPB or the other porphyrins was prevented by conducting all manipulations in the dark.

### PPIX-biotin (PPB) probe synthesis

The Fmoc protecting group was removed from Fmog-PEG Novatag resin (170 mg) using 20% piperidine in dimethylformamide (DMF). The protoporphyrin IX (PPIX) moiety was prepared by combining PPIX (25 mg) with 16.3 mg of 1-Hydroxy-7-azabenzotriazole N,N’-diisopropylcarbodiimide dissolved in 3 ml of N-methyl-2-pyrrolidone, which activated the carboxylic group and formed a symmetrical anhydride. Moieties were separated by a 13-atoms-long flexible linker that includes two polyethylene glycols attached by a carboxamide linker. The PPIX moiety mixture was added to the deprotected resin and left for 3 days at room temperature.

### NMR confirmation of probe, sample preparation and data acquisition and analysis of the spectra

The structure of PPB was confirmed with 2D NMR spectra: [1H, 1H]-TOCSY, [1H, 1H]-NOESY, [1H, 1H]-COSY, [15N, 1H]-HSQC and [13C, 1H]-HSQC 5 mg of PPB was dissolved in 500 ml of DMSO-d_6_. All the NMR data were acquired on a Bruker NMR spectrometer operating at 700 MHz for proton resonance under Topspin (version 4.1, Bruker, Germany) with a triple resonance TCI cryoprobe at 298 K. For the proton assignment of the probe molecule, PPB, two-dimensional (2D) [^1^H, ^1^H] COSY, TOCSY, NOESY (mixing time of 70 msec) experiments were performed. The carbon assignments were confirmed with a 2D [^1^H, ^13^C] HSQC and a 2D [^1^H, ^13^C] HMBC experiment. The nitrogen chemical shifts were confirmed using a 2D [^1^H, ^15^N] HSQC. All the spectra were processed using nmr Pipe and analyzed using Sparky. Since the product was dissolved in DMSO-d_6_ the exchange of the HN protons occur rapidly and hence several expected NOEs could not be observed in the spectrum, irrespective of using long mixing times. Though the PPB had decayed over a period of storage (few unassigned peaks in the spectra). Some of the quaternary carbon chemical shifts were derived from HMBC spectrum. Since the product was dissolved in DMSO-d_6_ the exchange of the HN protons are fast and hence several expected NOEs could not be observed in the spectrum, irrespective of using long mixing times. Chemical shift assignments of PPB (Fig. 4a–e and Supplementary Table 2 of Supplementary Information) show that the hydrogens at N8 and N7 demarcate the PEG linker connected to biotin (hydrogens at 49 and 56) and to PPIX [hydrogen at 32 (methyl)] respectively. The NOE’s observed from H32 (3.63 ppm, methyl) to HN7 (6.97 ppm) is an indicator of the PPIX linked to PEG in proximity, and the NOE’s from HN8 (6.95 ppm) to H81 (1.30 ppm), H49 (2.25 ppm) and H56 (0.765 ppm) show the connectivity between PEG and the Biotin moiety. These analyses agree with the proposed structure (Fig. 3b). However, the PPIX symmetry does not permit determination of whether the biotin is attached to either the C13 or C17 position of the PPIX propionate sidechain.

### PPB precipitation assays

Assays were performed using 500 µg of lysate per 50 µl of avidin beads. Lysates were first precleared by incubation with 50 µl of beads at room temperature for 30 min. Supernatants were transferred to fresh tubes and 40 nmol of PPB or biotin with only a cross-linker as a control were added to the tubes. The tubes were allowed to equilibrate in the dark for 30 min. For controls, to minimize non-specific interactions of proteins with PPB, we employed biotin, rather than PPIX as competitor as PPIX traps proteins at the high concentrations (above 1 mM) required to compete for binding to PPB. To the cleared lysate, 50 µl of beads in 50% slurry with PBS, 0.1% Triton X-100, was added and incubated an additional 45 min in the dark with occasional mixing. The beads were precipitated by centrifugation at 500 × *g* for 2 min and washed 8 times in 10 volumes of PBS/0.1% Triton X-100. Proteins were eluted in 2X Laemmli loading buffer with B-mercaptoethanol at 95 °C for 5 min before subsequent analysis.

### Protein digestion and peptide isobaric labeling by tandem mass tag (TMT)

Performed as previously described with slight modifications^38^. First, 2 µg of protein for each sample was electrophoresed on a short gel and stained by Coomassie blue before the protein concentration was determined using BSA as a standard. After protein estimation, gel bands were cut into smaller pieces for in-gel digestion. The gel bands were first washed with 50% acetonitrile, then reduced by adding 5 mM DTT at 37 °C for 30 min followed by alkylation with 10 mM iodoacetamide (IAA) for 30 min. in the dark at room temperature. Unreacted IAA was quenched with 30 mM DTT for 30 min. The gel bands were then washed, dried in a speed vacuum, and rehydrated with a buffer containing trypsin (Promega). Samples were digested overnight at 37 °C and acidified. The peptides were extracted in acetonitrile, dried in a speed vacuum, reconstituted in 50 mM HEPES (pH 8.5), and labeled with 11-plex Tandem Mass Tag (TMT) reagents (Thermo Scientific) according to manufacturer’s recommendations.

### Two-dimensional HPLC and mass spectrometry

The TMT-labeled samples were mixed equally, desalted, and fractionated over a 60-min gradient on an offline HPLC (Agilent 1220) by using basic pH reverse-phase liquid chromatography (LC). The fractions were dried and resuspended in 5% formic acid and analyzed by acidic pH reverse-phase LC-MS/MS analysis. The peptide samples were loaded on a nanoscale capillary reverse-phase C18 column (New objective, 75 um ID x ~ 25 cm, 1.9 µm C18 resin from Dr. Maisch GmbH) by HP and eluted over a 60-min gradient. The eluted peptides were ionized by electrospray ionization and detected by an in-line Orbitrap Fusion mass spectrometer. The mass spectrometer is operated in data-dependent mode with a survey scan in Orbitrap (60,000 resolution, 2 × 105 AGC target and 50 ms maximal ion time) and MS/MS high-resolution scans (60,000 resolution, 2 × 105 AGC target, 200 ms maximal ion time, 38 HCD normalized collision energy, 1.5 *m*/*z* isolation window with 0.2 *m*/*z* offset, and 20 s dynamic exclusion).

### Identification and quantification of proteins with JUMP software suite

Proteins were identified and quantified by using the JUMP proteomics software suite to evaluate the false-discovery rate (FDR). All original target protein sequences were reversed to generate a decoy database that was concatenated to the target database. The target protein database was generated by combining both human and mouse protein sequences downloaded from UniProt, with contamination proteins added. Major parameters included precursor and product ion mass tolerance (±15 ppm), full trypticity, static mass shift for the TMT tags (+229.16293), carbamidomethyl modification of 57.02146 on cysteine, dynamic mass shift for Met oxidation (+15.99491), maximal missed cleavage (*n* = 2), and maximal modification sites (*n* = 3). Putative peptide-spectrum matches (PSMs) were filtered by mass accuracy and then grouped by precursor ion charge state and filtered by JUMP-based matching scores (Jscore and ΔJn) to reduce the FDR below 1% for proteins. If one peptide could be generated from multiple homologous proteins, based on the rule of parsimony, then the peptide was assigned to the canonical protein form in the manually curated Swiss-Protein database.

### Tandem mass tag-based quantification analysis

TMT-based quantification analysis was performed in the following steps. First, TMT reporter-ion intensities of each PSM were extracted. Second, the raw intensities were corrected based on isotopic distribution of each labeling reagent (e.g., TMT126 generates 91.8%, 7.9% and 0.3% of 126, 127, 128 *m*/*z* ions, respectively). Third, PSMs of very low intensities (e.g., minimum intensity of 1000 and median intensity of 5000) were excluded. Fourth, the mean-centered intensities were calculated across samples (e.g., relative intensities between each sample and the mean). Fifth, protein or phosphopeptide relative intensities were summarized by averaging related PSMs. And sixth, the relative intensities were multiplied by the grand-mean of the three most highly abundant PSMs to derive protein or phosphopeptide absolute intensities.

### Adjustment of loading bias by species-specific peptides

To minimize technical variation, loading-bias correction has become a crucial step for quantitative proteomics analysis, based on the assumption of equal input amount across all samples. However, this assumption may not be valid for precipitation experiments. To overcome this, we pooled both human and mouse cell lines into one 11-plex TMT experiment so that species-specific peptides were leveraged to correct loading bias across samples, with the following assumption: mouse-specific peptides in human cell lines should have TMT signals similar to those of blank samples, whereas the opposite should be true for human-specific peptides. Briefly, all quantified peptides were separated into three categories: human-specific peptides, mouse-specific peptides, and peptides shared between the two species. To adjust loading bias in a human cell line, we defined the loading-bias normalization factor as median (TMT signal across PSMs from mouse-specific peptides in that cell line)/median (TMT signal across PSMs from mouse-specific peptides in the 1st blank sample). A similar strategy was implemented to adjust loading bias for mouse cell lines. The corrected data were used for all downstream analyses (e.g., differential expression, network analysis).

### Statistical analysis for identification of PPIX interaction candidates

Four cell lines (two human and two mouse) with duplicates and three blank negative control samples were analyzed. To avoid bias of protein quantification due to species-specific peptides, only homologous peptides shared between human and mouse were used for protein quantification. For each of the four cell lines, interaction candidates were identified by comparison to the three blank samples via one-tailed *t*-test. The four results (i.e., the *p* value for individual tests) were combined into one integrative *p* value by using Fisher’s method, and the resulting combined *p* value was then converted to FDR (for multiple tests correction) by using the BH method. In addition, the measurement variation was based on the replicated measurements of the samples. The relative expression (i.e., ratio) between replicates for each protein was calculated. The ratios of all proteins from the replicates were fitted with a Gaussian distribution to estimate expected mean and standard deviation (null SD). Proteins showing a combined FDR < 0.01 with log2 fold change larger than 3 null SD were considered to be interaction candidates. We used Significance Analysis of Interaction (SAINT) software for scoring PPB-interacting proteins (Supplementary Data 6). For each cell line, three independent cell lysates were prepared, incubated with PPB, and controls included: PEG-biotin alone, and PPB with excess PEG-biotin. To stringently quantify PPB interactions, sample loading bias was corrected by using species-specific peptides and PPB-interacting proteins determined by SAINT analysis, allowing for accurate quantification. After removal of negative control samples, 543 discrete candidates were identified as PPB interaction proteins (FDR < 0.01 and *z*-score >3; see Supplementary Data 1, tab Data Candidates). These PPB-interacting proteins localized to multiple subcellular compartments with the largest portion (35%) being cytosolic (Fig. 5b). Using immunoblotting we were able to validate that ANT1 and ANT2 are pulled down by PPB (Supplementary Fig. 3a) which is consistent with their proposed role as PPIX transporters.

### Co-expression and protein-protein interaction (PPI) network analysis

The analysis was performed by using JUMP software as previously described. Briefly, the identified PPIX interaction candidates were clustered into eight co-expression clusters by using the WGCNA R package^49^. Proteins in each cluster were then superimposed onto a composite PPI database by combining STRING-identified PPI data (STRING version 11.5 STRING version 11.5: protein-protein interaction networks, integrated over the tree of life). Modules in each protein cluster were defined by calculating a topologically overlapping matrix for the PPI network and modularizing such a network into individual modules by using the hybrid dynamic tree-cutting method. Modules were annotated by using three pathway databases, including Gene Ontology (GO), KEGG, and Hallmark, by Fisher’s exact test, and visualized by using Cytoscape (version 3.7.2).

#### Pathway and cluster analysis

Multiple pathway analysis programs and gene ontology databases (KEGG, GO, and Hallmark, etc.) were interrogated to identify pathways common to PPB-binding proteins (Supplementary Data 2, see tabs Enrichment function, Enrichment process, Keyword, Pfam, KEGG, component and RCTM (reactome)). String database (version 11.5), Cytoscape (www.cytoscape.org; V3.7.2) and Enricher (https://maayanlab.cloud/Enrichr/) were used to extend analysis. Given that PPIX formation elevated. To identify redox pathway constituents, we queried databases GO and KEGG as well as Pubmed and Google scholar and queried “redox” to discover a subset of redox proteins that interacted with PPB (Supplementary Data 4) noting that three of the six peroxiredoxin family members, PRDX1, PRXD2, and PRDX3, bound PPB. An unweighted gene-clustering analysis^49^ revealed eight distinct clusters among the PPB-binding proteins^50^ (Supplementary Fig. 4). Although many proteins were in common among the various cell lines, cluster 1 segregated the NIH3T3 cells from the other cell lines.

#### DepMap screening

The DepMap portal (depmap.org/portal/) was queried for cell dependency using ferrochelatase (FECH) as a search term. Of several cancer cells showing relative reliance on the enzyme, the T-lymphoblastoid cancer cell line, Jurkat, was identified as having a frame shift deletion in FECH. Subsequent testing indicated that this cell line was sensitive to the cytotoxic effects of PPIX.

### Ferrozine iron assay

Cells were seeded at 1 × 10^5^ per well of a 24-well plate and treated as described in the figure legend. Cells were treated with 100 μl of 10 mM HCl and 100 μl of a solution of equal volumes of 1.4 M HCl and 4.5% potassium permanganate and incubated for 1 h at 60 °C within a fume hood. Then, 30 µl of detection reagent (6.5 mM ferrozine, 6.5 mM neocuproine, 2.5 M ammonium acetate, and 1 M ascorbic acid) was added to each tube. After a 30-min incubation, the solution was transferred into a well of a 96-well plate, and the absorbance was measured at 550 nm.

### Heme and porphyrin determination

Cells were pelleted and lysed in acetone acidified by 20% 1.6 N HCl for 5 min at room temperature and then centrifuged (21,000 × *g*) for 5 min at 4 °C. Then, 100 µl of supernatant was injected into the HPLC system (Shimadzu SIL20AC), and heme and PPIX were separated on a Supelco 125 × 4.6-mm C18 column (3 μm) by applying a 30–66% linear gradient mobile phase over 5 min followed by a 66–90% linear gradient over 20 min. Heme absorbance was read at 400 nm wavelength, whereas porphyrin fluorescence was measured at wavelengths of 395 nm (excitation) and 630 nm (emission). Results were quantified by extrapolation to known quantities of external standards and normalized to cell number.

### Electron microscopy

Samples for electron microscopy were fixed as monolayers in a mixed aldehyde fixative in cacodylate buffer, then scraped and pelleted for further processing. Samples were post-fixed in osmium tetroxide and contrasted with uranyl acetate. Following dehydration in an ascending series of alcohols, samples were transitioned into EMbed812 resin with propylene oxide as the transitional solvent. Once in 100% resin, samples were polymerized overnight at 80 °C. Samples were sectioned at ~70-nm thickness on a Leica (Wetzlar, Germany) UC-7 ultramicrotome and examined in a Thermo Fisher Scientific F20 transmission electron microscope. Images were captured on an AMT camera system (Woburn, MA). Unless otherwise stated, all reagents were sourced from Electron Microscopy Sciences (Hatfield, PA).

### Mitochondrial morphology

Images captured by electron microscopy (at least 16 representative fields with visible mitochondria) were analyzed for each treatment set of 3T3 cells: untreated or treated for 24 h with ALA (200 µM), succinylacetone (250 µM) or with both. To avoid bias, samples were masked before scoring by the St. Jude Electron Microscopy Core. Mitochondria in each field (16–30 fields per treatment) were scored using the following scale: 0—normal morphology mitochondria, 1—no defect except a few cristae not extending across the mitochondria or minor variations in direction of cristae, 2—many mitochondria with missing cristae, altered cristae direction, or highly unique/unusual mitochondrial shape, 3—severely impaired mitochondria: most or all cristae missing or strongly altered, and/or mitochondria with membranes ruptured.

### Cell viability

Cells were seeded at either 2.5 × 10^3^ or 10^4^ cells per well of 96-well plates in 100-µl volume and treated with compounds as indicated in legends. Cell viability was determined using an equal volume of Cell Titer Glo reagent (Promega) added directly to the media. After 5 min of mixing, the lysed cells in media/reagent solution were transferred to white plates (Thermo), and luminescence was determined in the Synergy H4 plate reader. Fluorescence was normalized to controls within each treatment group.

### Glutathione

Cells were seeded at 10^4^ per well of 96-well plates in 100-µl volume, and experimental wells were treated for 24 h with 200 µM ALA alone or with other agents as indicated in legends. Total glutathione or GSSG was then measured by using the GSH/GSSH Glo kit (Promega) according to the manufacturer’s instructions. The fraction of oxidized glutathione was determined for each treatment group and normalized to untreated controls.

### Glutathione peroxidase

Gpx activity was measured by coupled assay with glutathione reductase. Reaction mixture was 200 µM NADPH, 1 mM reduced glutathione, 1 mM sodium azide, 1 mM EDTA, and 3 units/ml of glutathione reductase in 50 mM Tris (7.4). Lysates were prepared by sonication in 50 mM Tris (7.4) with 0.1% Triton X-100 followed by centrifugation at 21,100 × *g* for 5 min to clear the lysate. Background was measured following the oxidation of NADPH at 340 nm in the presence of lysate alone. Reactions were started by bringing the reaction mix to 1 mM with cumene hydroperoxide and changes in absorbance A340 were recorded for 20 min at 5-min intervals. Specific activity was expressed as nmol min^−1^ mg^−1^.

### Flow cytometry

Cells were seeded at 2 × 10^5^ per well in 12-well plates and treated as indicated in legends for the times indicated. Cells remained in the dark with all fluorescent indicators followed by trypsinization and one wash with 1X PBS (Gibco). The indicators employed and fluorescence excitation/emission wavelengths were as follow: MitoSOX Red, mitochondrial superoxide indicator, 2 µM, (510/580 nm); DCFDA, general oxidative stress indicator, 5 µM, (485/535 nm); C_11_-Bodipy, lipid peroxidation indicator, 1 µM (488/545 nm); TMRE, mitochondrial potential indicator, 200 nM, (488/561 nm); Phen Green SK, cellular iron indicator, 10 µM, (400/510). Positive controls were added 30 min prior to staining and included antimycin A, 1 µM, for MitoSOX Red; 0.5 mM hydrogen peroxide for DCFDA and C11-Bodipy; FCCP, 10 µM for TMRE; and 50 µM ferrous ammonium citrate for Phen Green SK. Background was subtracted using unstained cells, and mean fluorescence intensity of viable cells was normalized to stained controls, except for Phen Green. Because elevated iron concentration caused by Phen Green results in reduced fluorescence, these data are presented as inverse fluorescence.

### PRDX3 dimerization

NIH3T3 cells were trypsinized and suspended at 10^4^ per µl in phosphate-buffered saline at room temperature. Cells were brought to the concentration indicated with hydrogen peroxide or PPIX and equilibrated for 5 min before lysis by addition of Trident 6X Laemmli buffer. Samples were immediately sonicated for 5 s, then those either reduced to monomer by treatment with β-mercaptoethanol or left untreated to show dimer formation. Following 2 min incubation at 90 °C, lysates were centrifuged 5 min at 21,000 × *g* and analyzed by immunoblotting for PRDX3.

### Cell surface labeling

Cells were seeded at 4 × 10^5^ density in 10 cm dish. When cells reached 90% confluency, they were treated with 200 µM ALA and 250 µM SA for 24 h and then washed with PBX and harvested by trypsinization. The cells were then incubated with 0.25 mg/ml of EZ-Link Sulfo-NHS-SS-Biotin (Pierce) solution in PBS at 4 °C for 30 min, with gentle mixing in a rotator. Excess labeling reagent was quenched by incubating the cells in 5 mM Tris (pH 7.6). The cells were lysed in RIPA lysis buffer containing 1x protease inhibitor cocktail (Roche), 0.2 M phenylmethylsulfonyl fluoride (PMSF), 1M N-Ethylmaleimide (NEM) and 20 mM MG132. Protein quantity in the cell lysate was measured by BSA protein assay. Cell lysate containing 100 μg protein was incubated with 33 μl of streptavidin-agarose beads (Pierce) for 16–18 h on a rotator at 4 °C. The biotinylated surface proteins attached to the streptavidin agarose beads were eluted by boiling at 95 °C for 5 min. The total eluates were analyzed by immunoblotting.

### Gene knockdown

NIH-3T3 cells were seeded in growth media at 2 × 10^5^ cells per well of a 6-well plate the day prior to siRNA transfection. Control and On-Target Plus siRNA pools directed against each target protein were obtained from Horizon/Dharmacon (Cambridge, UK), ferritin (L_047068_00_0020), Prdx3 (L_043352_01), and transferrin receptor (L_055550_01). Transfections for each well were performed with the desired siRNA in 150 µl of media and combined with equal volume of OPTIMEM containing 9 µl Lipofectamine 2000. The final siRNA concentrations were 20 nM for ferritin and Prdx3 and 50 nM for the transferrin receptor. After 48 h cells were split into 96-well plates as described in experiment legends or lysed for Western blotting. After attachment, cells were either left untreated or treated with a combination of 20 µM ferrostatin-1 and various concentrations of ALA. After an additional 48 h viability was assessed using Cell Titer Glo reagent (Promega, Madison, WI) as per the manufacturer’s instructions.

### Reporting summary

Further information on research design is available in the Nature Portfolio Reporting Summary linked to this article.

## Supplementary information

Supplementary Information

Description of Additional Supplementary Files

Supplementary Data 1

Supplementary Data 2

Supplementary Data 3

Supplementary Data 4

Supplementary Data 5

Supplementary Data 6

Supplementary Data 7

Reporting Summary

## Data Availability

Proteomic data will be available through the Dryad data depository via 10.5061/dryad.mkkwh712t. Numerical source data for all charts and graphs is available in Supplementary Data Table 1. Original images for figures may be found, as Supplementary Figs. 12–25, in the Supplementary Information file. Any other data will be provided upon request of the corresponding author.

## Code availability

Analysis was performed using custom JUMP software. Jump code is freely available at https://www.stjude.org/research/labs/peng-lab-junmin/jump-software.html.
