# Supplementary material for: A PPIX-binding probe facilitates discovery of PPIX-induced cell death modulation by peroxiredoxin
Source: Commun Biol. 2023 Jun 24;6:673. doi: 10.1038/s42003-023-05024-5 (PMC10290680; doi:10.1038/s42003-023-05024-5)
Supplement: Supplementary file 1 — Supplementary Information [file 42003_2023_5024_MOESM1_ESM.pdf]

Supplementary Table 1. Antibodies

Name	Source	Catalog Number
Ferritin	Abcam	AB 75973
$Na^{+}K^{+}$ ATPase	Novus	NB 300-146SS
Prdx1	Invitrogen	LF-MA0073
Prdx2	Invitrogen	LF-MA0144
Prdx3	Abnova	MAB2725
TfR	Zymed	13-6800

Supplementary Table 2. NMR Shifts

Group	Atom	Shift	SDev	Group	Atom	Shift	SDev
B1	C1	53.171	0.015	P1	C45	67.706	0.045
B1	C10	161.686	0.022	P1	C46	68.876	0.01
B1	C2	56.24	0.044	P1	C47	69.363	0.026
B1	C3	70.156	0	P1	C48	68.385	0.021
B1	C4	59.067	0.008	P1	C49	36.126	0.033
B1	C5	25.648	0.02	P1	C50	11.868	0
B1	C6	27.089	0	P1	C51	29.123	0.026
B1	C7	25.591	0.019	P1	C52	69.122	0
B1	C71	1.504	0.002	P1	C53	69.123	0
B1	C8	35.524	0	P1	C56	29.73	0.007
B1	H11	4.418	0.003	P1	C9	172.384	0.015
B1	H21	4.286	0.005	P1	H111	6.244	0.003
B1	H3	2.875	0.005	P1	H112	6.481	0.006
B1	H41	3.317	0.002	P1	H121	8.53	0.018
B1	H42	2.862	0.003	P1	H16	10.381	0.005
B1	H51	1.709	0.002	P1	H20	3.77	0.006
B1	H61	1.398	0.004	P1	H22	10.302	0.006
B1	H71	1.493	0.003	P1	H25	3.64	0.008
B1	H81	2.023	0.003	P1	H28	10.292	0.003
B1	HN1	6.672	0.001	P1	H32	4.353	0.005
B1	HN2	6.766	0.003	P1	H34	10.289	0.006
B1	N1	91.286	0	P1	H36	3.016	0.003
B1	N2	83.286	0	P1	H37	3.24	0.003
P1	C11	121.832	0	P1	H39	7.079	0.006
P1	C12	130.696	0	P1	H40	7.341	0.004
P1	C13	135.62	0	P1	H41	1.693	0.004
P1	C14	143.86	0	P1	H42	4.094	0.005
P1	C15	137.565	0	P1	H44	2.909	0.002
P1	C16	98.266	0	P1	H45	2.442	0.003
P1	C19	129.292	0	P1	H46	2.254	0.003
P1	C20	13.231	0	P1	H47	2.586	0.004
P1	C21	135.929	0	P1	H48	3.193	0.003
P1	C22	97.866	0	P1	H49	2.972	0.003
P1	C25	11.858	0	P1	H50	3.114	0.935
P1	C28	98.312	0	P1	H51	1.149	0.003
P1	C32	22.119	0.011	P1	H52	2.249	0
P1	C34	97.448	0	P1	H53	2.573	0
P1	C36	38.699	0	P1	H56	1.492	0.002
P1	C37	37.184	0	P1	HN4	9.499	0.006
P1	C38	174.647	0	P1	HN6	8.546	0.005
P1	C39	129.379	0.09	P1	HN7	7.692	0.002
P1	C40	118.779	0.004	P1	HN8	7.68	0.004
P1	C41	25.69	0.001	P1	N4	103.985	0
P1	C42	64.131	0.016	P1	N6	107.255	0
P1	C43	154.072	0	P1	N7	118.693	0
P1	C44	36.056	0.025	P1	N8	118.032	0

Supplementary Fig. 1. Design and testing of the PPIX-biotin (PPB) probe. A) Workflow for the development of PPIX-biotin probe and assay for subsequent pulldown experiments and analysis. **B**) Principle for testing albumin binding by PPB: albumin-bound PPB will pass through size-exclusion gel while free probe will be retained. **C**) PPB from 12.5-100 µM is retained by G25 column.(single individual samples) **D**) Protein bound PPB passes through the column. (n=4 samples from single experiment, mean ± SEM)

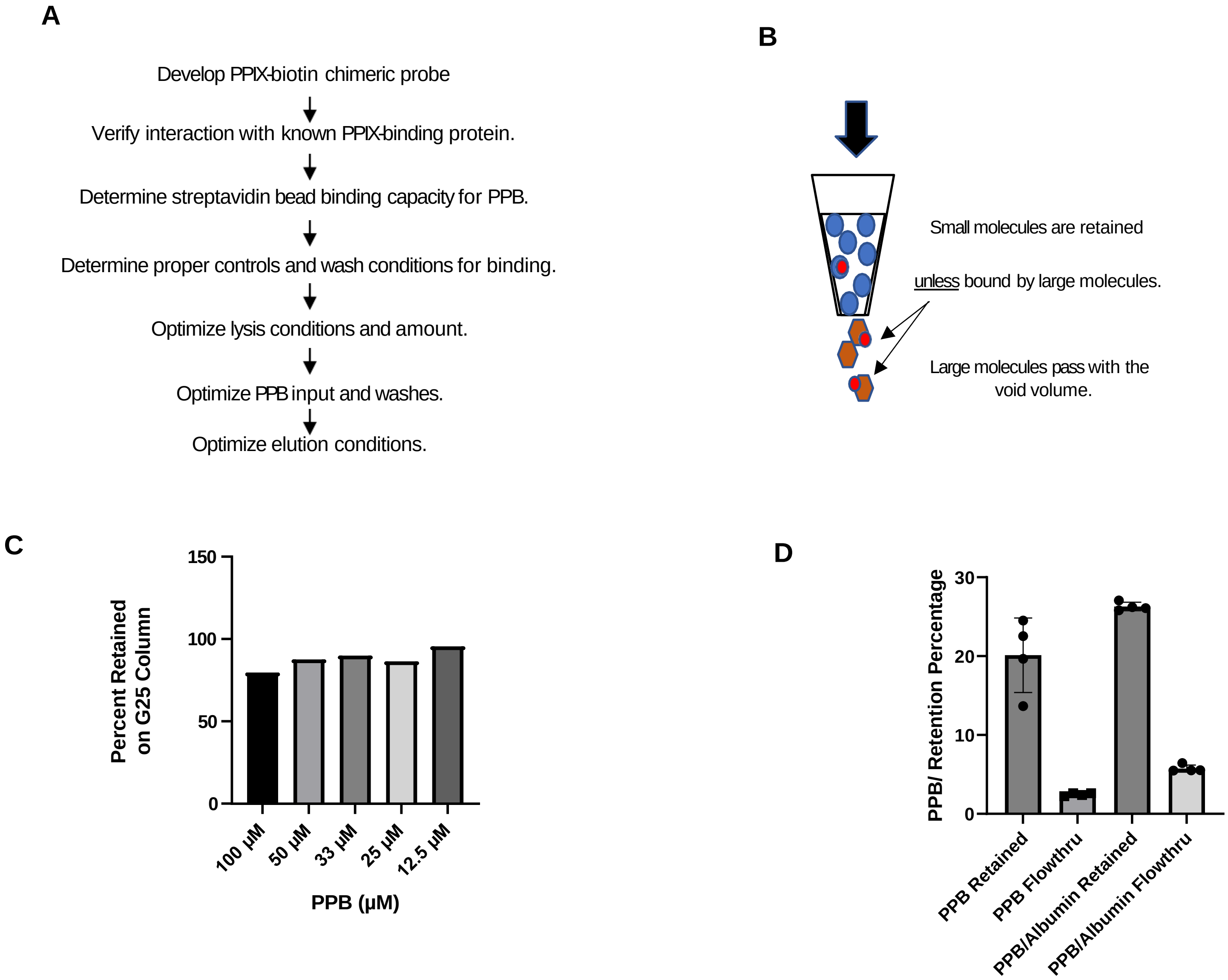

Supplementary Fig. 2. **PPB Pulldown Development** a) Photographs showing color of beads following the washes indicated, demonstrating accumulation of PPIX with PPB coincubation, but not alone. (n=2 independent experiments) b) Model for PPB pulldown competition experiments. c) Silver staining demonstrating protein pulldown under competition with PPIX, biotin and PEG-biotin and d) quantitation of lane density. (n=2 independent experiments)

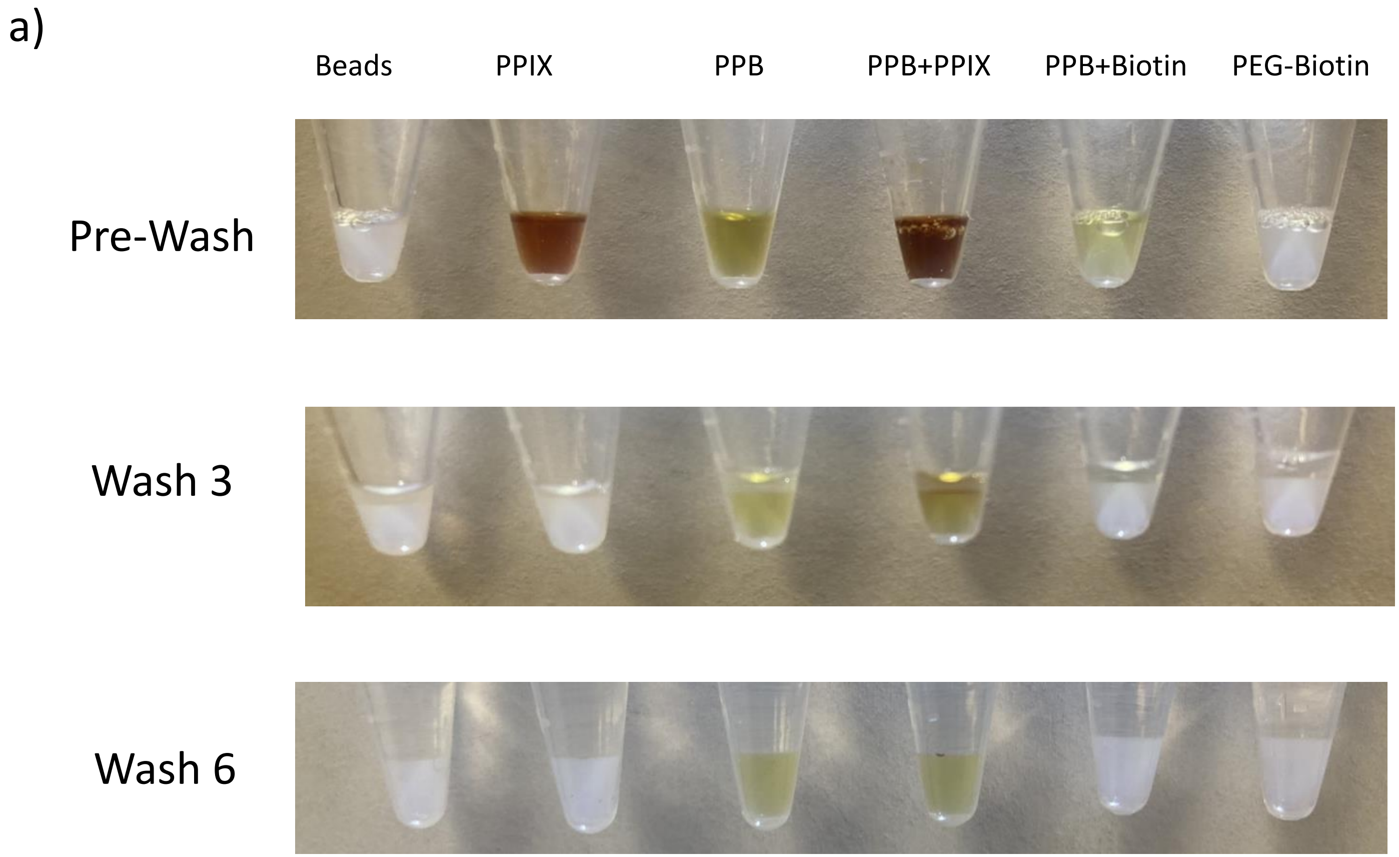

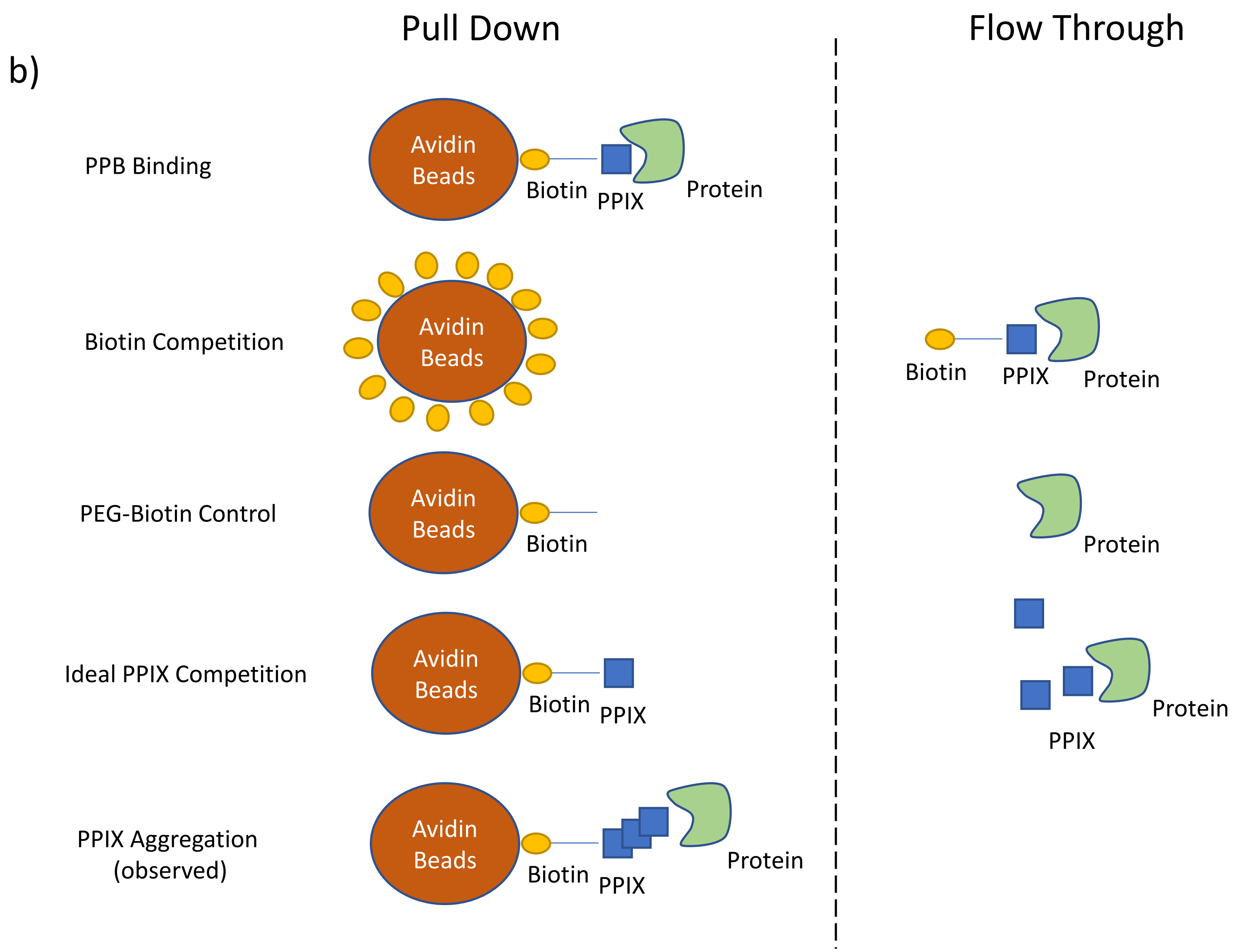
b)
Pull Down
Flow Through
PPB Binding
Avidin Beads
Biotin
PPIX
Protein
Biotin Competition
Avidin Beads
Biotin
PPIX
Protein
PEG-Biotin Control
Avidin Beads
Biotin
Protein
Ideal PPIX Competition
Avidin Beads
Biotin
PPIX
Protein
PPIX
PPIX Aggregation (observed)
Avidin Beads
Biotin
PPIX
Protein

c)

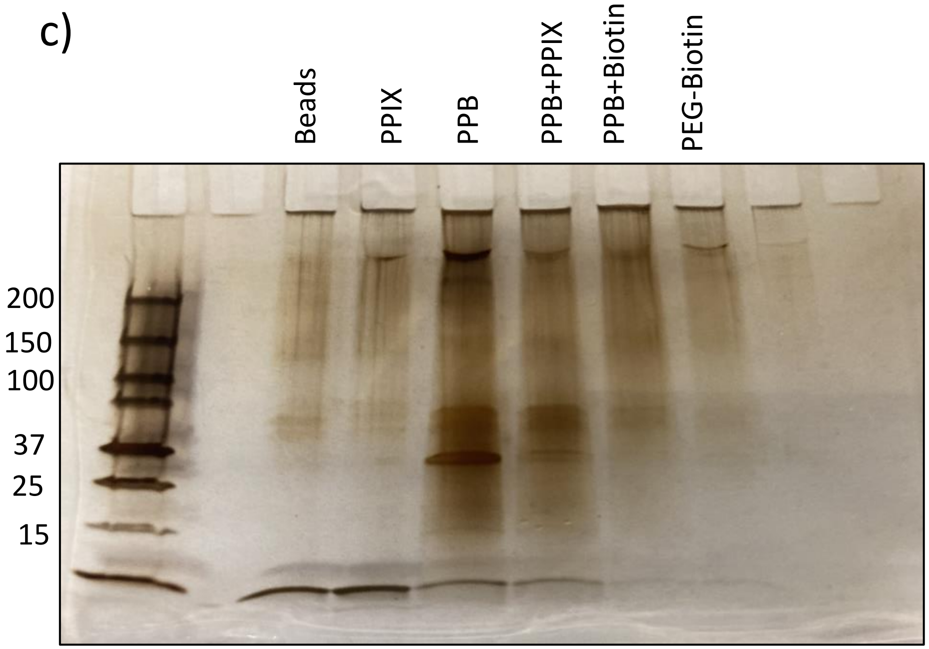

Beads
PPIX
PPB
PPB+PPIX
PPB+Biotin
PEG-Biotin
200
150
100
37
25
15

d)

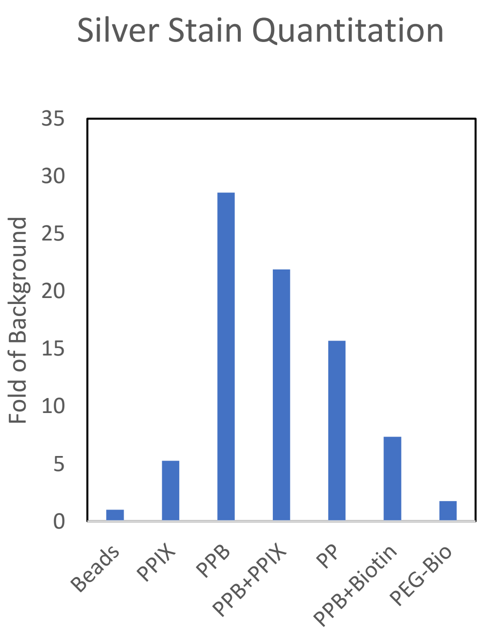

Silver Stain Quantitation
Fold of Background
35
30
25
20
15
10
5
0
Beads
PPIX
PPB
PPB+PPIX
PP
PPB+Biotin
PEG-Bio

Supplementary Fig. 3. Immunoprecipitation confirmation of Proteins Identified in PPB Proteomic Screen a) 100 mg U251 lysate (input) was precleared 2X with washed streptavidin beads.The supernatant (post-clearance) was incubated with 20 nM PPB(ox) in the dark then bound with 100 ml of fresh beads. Supernatant (sup) was removed and beads washed 6X in PBS/0.1% TX100 and beads and beads extracted in Laemmli buffer (pulldown) for 5 min at 95 C. (Controls: Bk, biotin-linker only; +, PPB; -, PPB with excess biotin). SDHA/B/C were from a single membrane while ANT1/2 were taken from separate membranes. (n=4 for SDHA, n=2 for other proteins) b) Bovine heart mitochondria treated with 50 µM or 100 µM PPIX and succinate dehydrogenase activity was measured for 1 minute following addition of substrate (n=3 independent experiments, mean ± SEM).

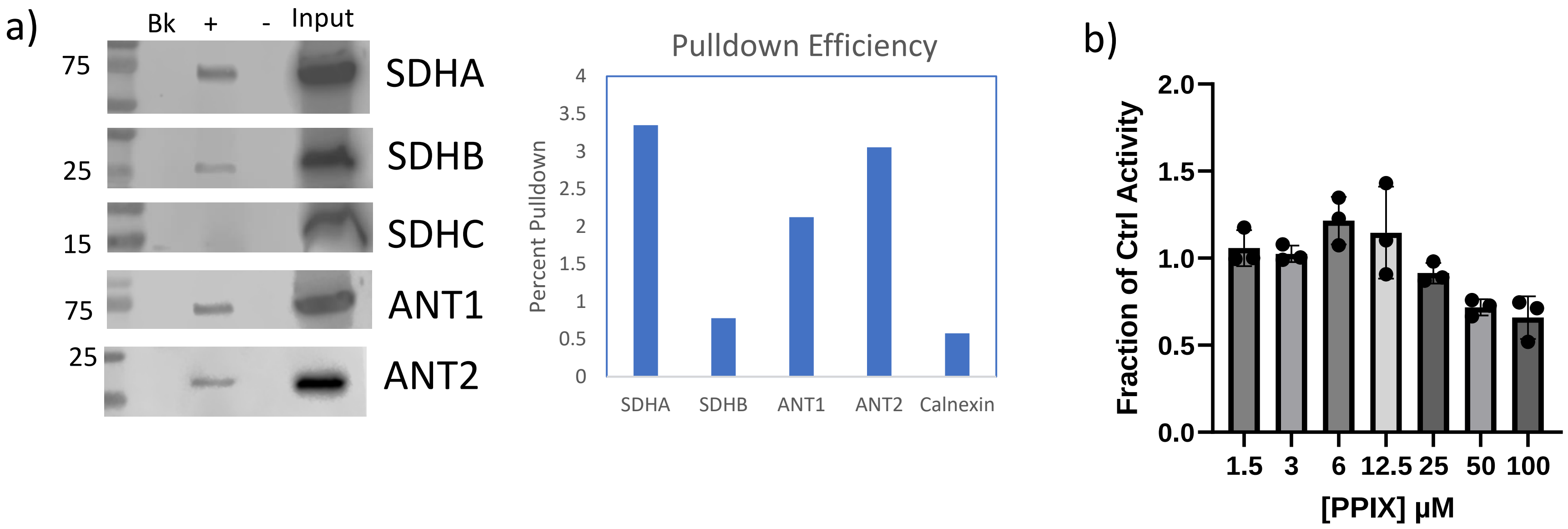

Supplementary Fig. 4. Relative level of protein clusters among cell lines. WGCNA (Weighted gene co-expression network analysis) of TMT quantitative proteomics data by PPIX pulldown identified eight distinct co-expression patterns across cell lines. Each dot represents one protein, with the number of proteins within each cluster indicated in parentheses.

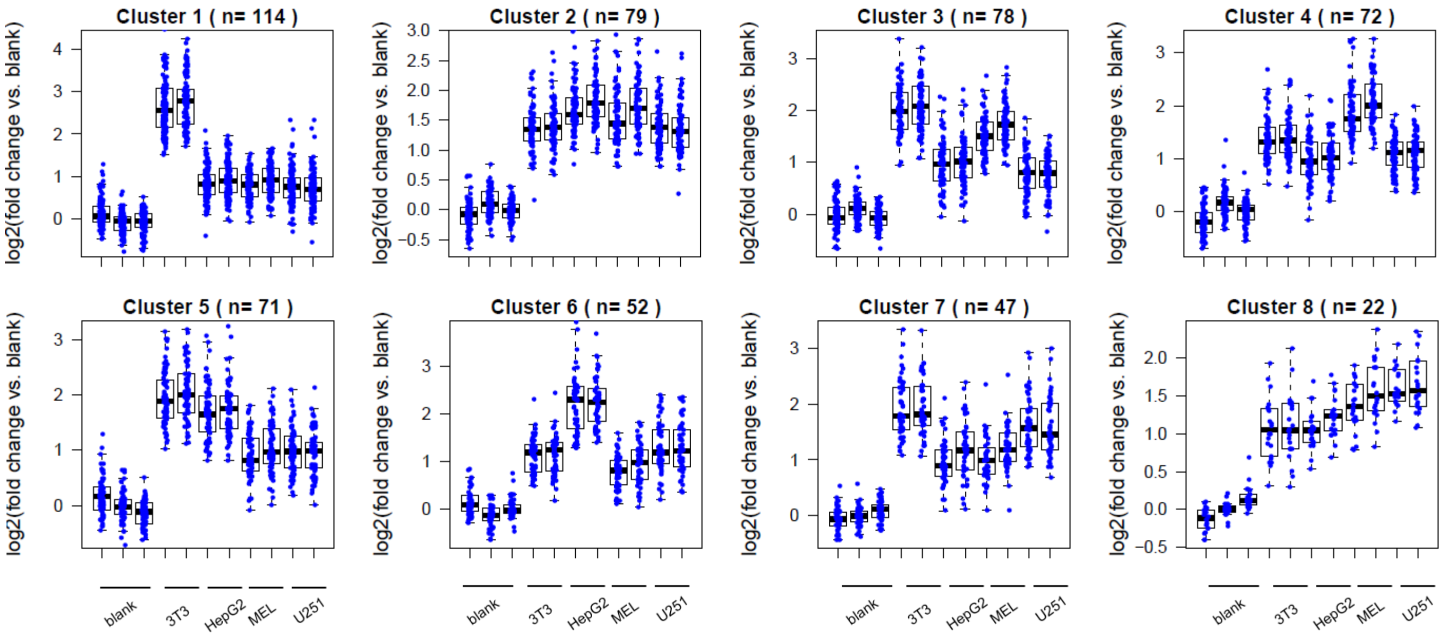

Supplementary Fig. 5. Succinylacetone does not rescue erastin toxicity. NIH3T3 were treated for 48 hours with varying concentrations of erastin in the presence or absence of 250 µM succinylacetone. (n=3 independent experiments, mean ± SEM)

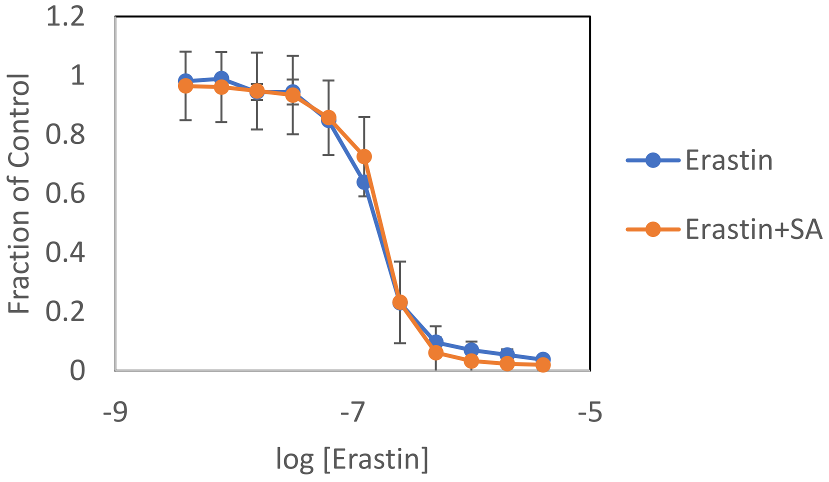

Supplementary Fig. 6. ALA treatment of NIH-3T3 cells does not alter cell cycle. NIH3T3 cells were treated with 200 µM ALA at a) 24 hours b) 48 hours or c) 72 hours progression, as determined by FACS by using propidium iodide. (n=2 independent samples from a single experiment, mean ± SEM)

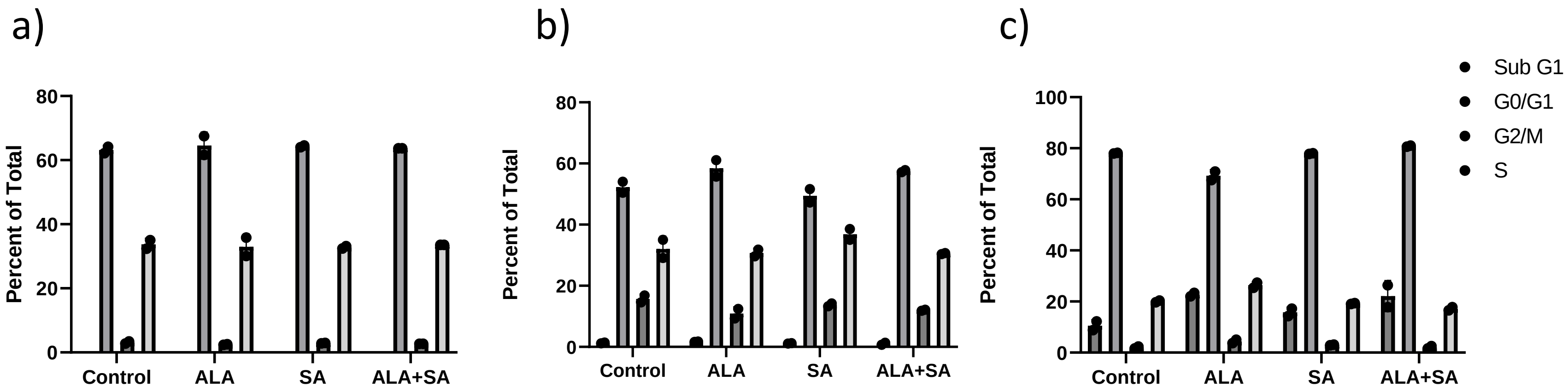

Supplementary Fig.. 7. Endocytosis inhibitor effect on erastin and ALA toxicity in NIH3T3. A NIH3T3 cotreated with 250µM ALA and endocytosis inhibitors Dynasore (15 µM), Pitstop (20 µM) or sucrose (100 mM) show increased viability at 48 hours relative to ALA treatment alone. (n=3 independent experiments) B). Cotreatment with Dynasore (10µM), but not other inhibitors of endycytosis, sucrose and pitstop, rescued erastin toxicity in NIH3T3. (n=2 independent samples, representative of 3 independent experiments, mean ± SEM) C). Treatment with ALA and sucrose increase membrane associated ferritin alone and in combination.

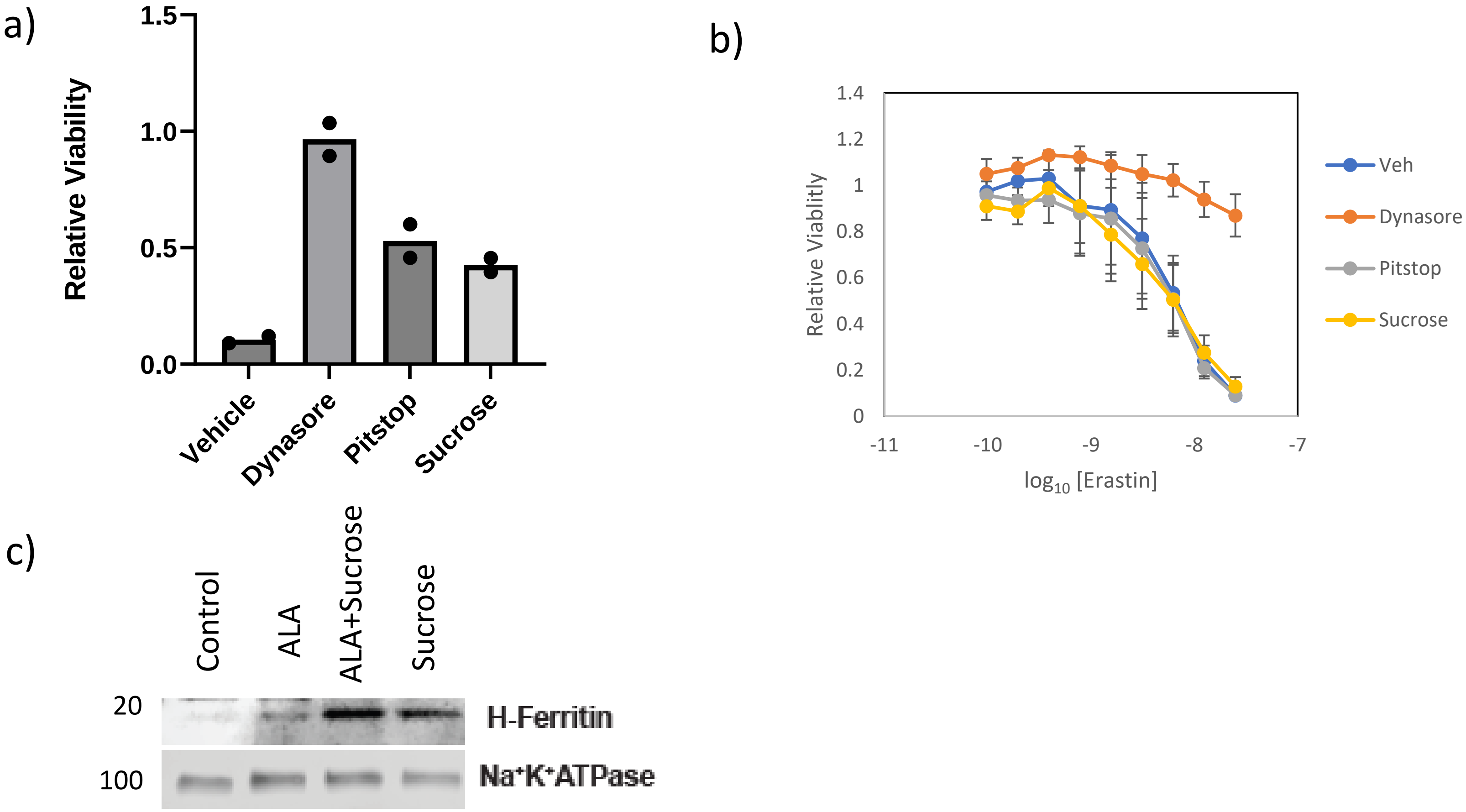

Supplementary Fig. 8. Analysis of combined effects of ALA & Erastin cotreatment. Erastin and ALA cotreatment demonstrate antagonism by RSM Analysis. (n=4 datasets from independent experiments for analysis).

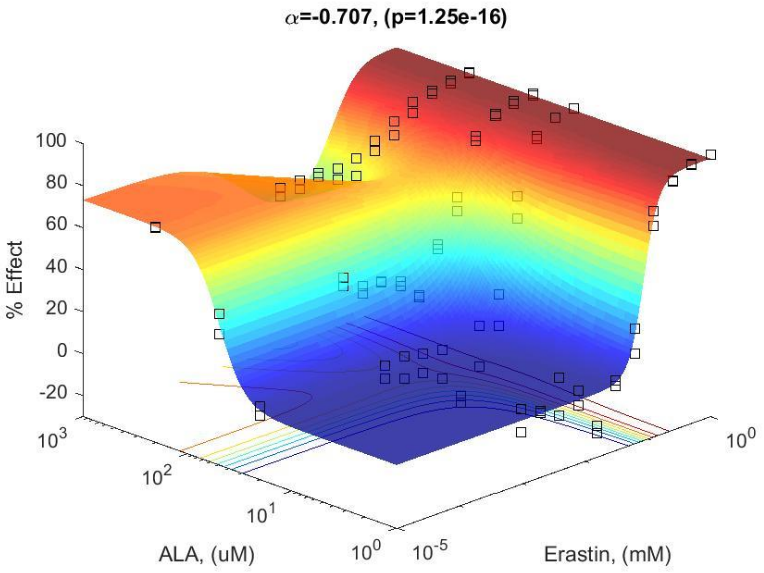

Supplementary Fig. 9. Common Heme and PPIX binding proteins by Venn diagram

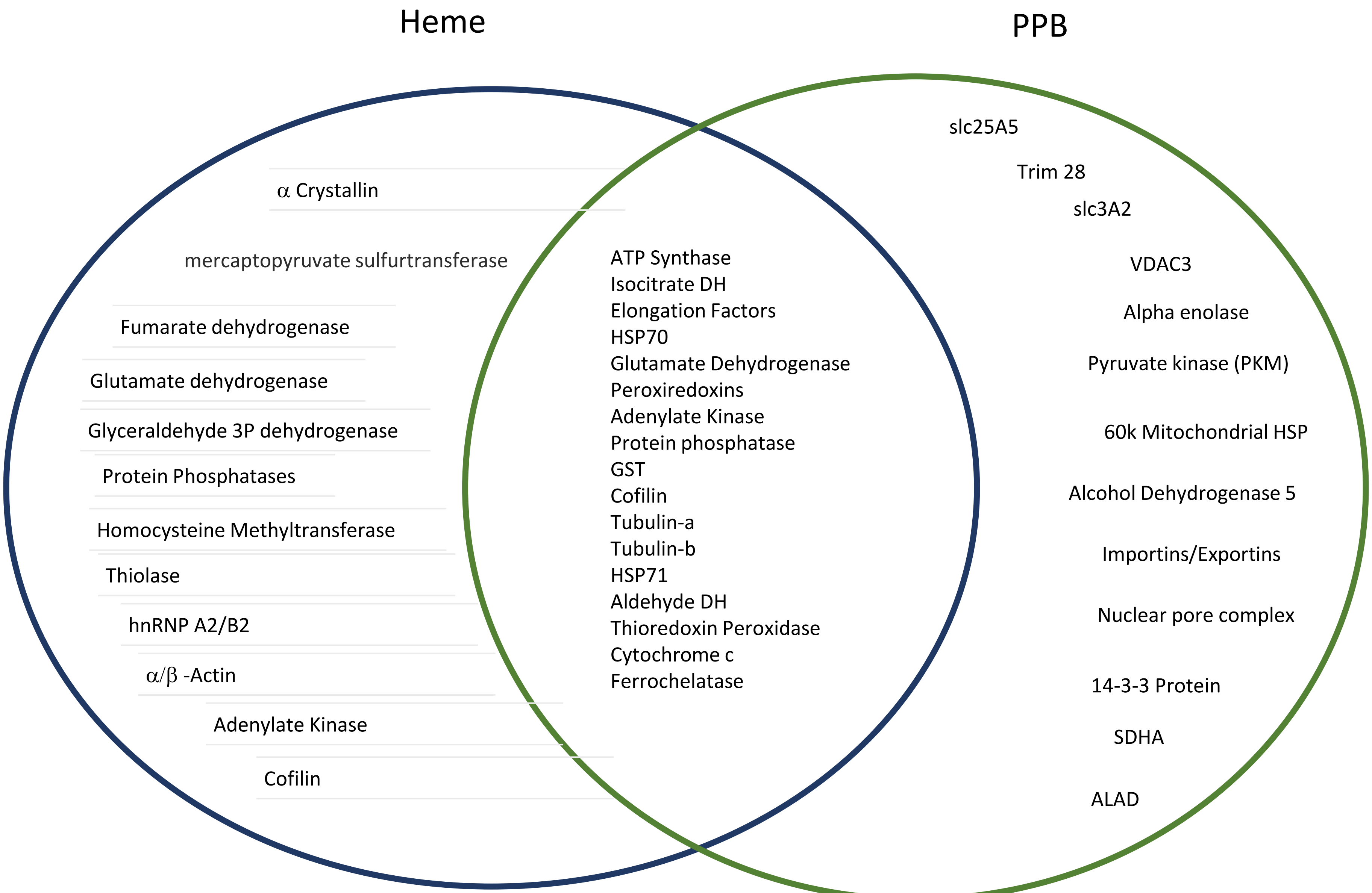

Supplementary Fig. 10. Ferrostatin, but not GSK or ZVAD, rescue viability in ALA treated NIH3T3.
ALA treatment cotreatment with ZVAD (10µM), GSK872 (3µM), necrosulfonamide (5 µM) or ferrostatin-1 (10 µM). (n=2 independent data points, representative of 2-5 independent experiments for all inhibitors, mean ± SEM)

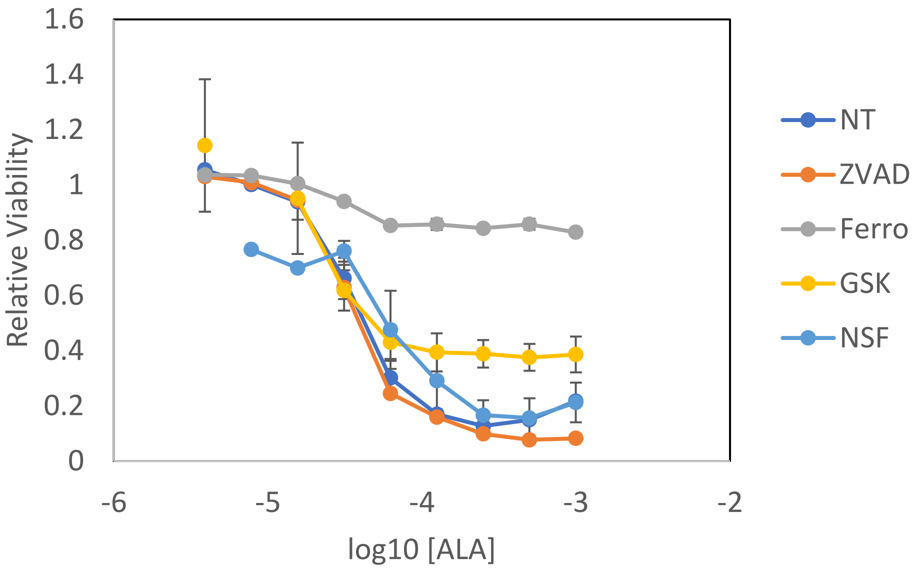

Supplementary Fig. 11 Inhibition of ABCC1 with MK571 does not provide rescue for ALA toxicity in NIH3T3. Cotreatment of NIH3T3 with MK571 (10µM), sufficient(to inhibit ABCC1 activity does not increase viability for ALA treatment at 48 hours. (n=2 independent experiments (n=2 independent samples, representative of 2 independent experiments, mean ± SEM))

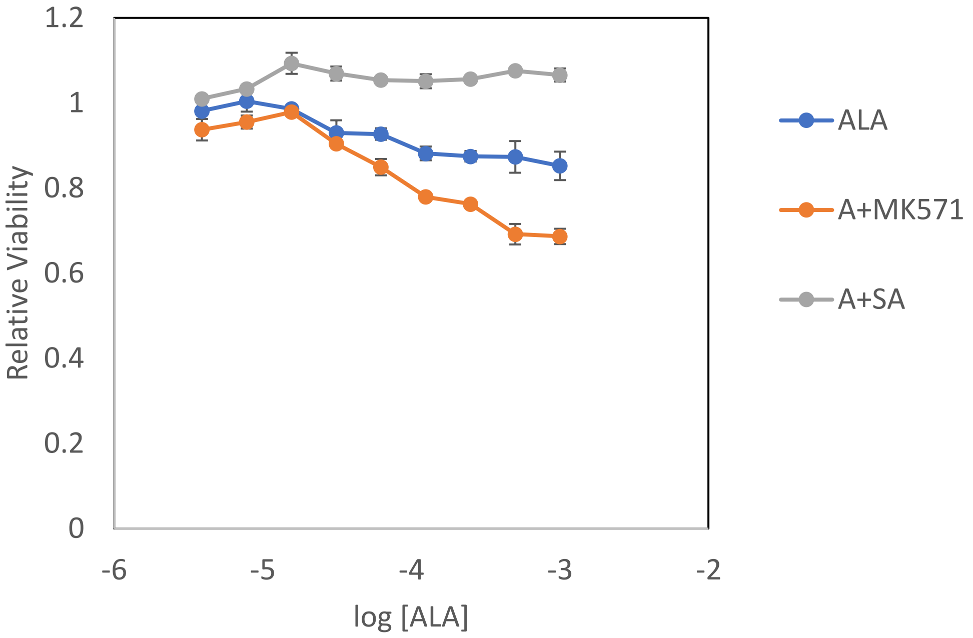

Supplementary figure 12: Unedited photos for Figure 2a, electron micrographs of mitochondria in treated NIH3T3.

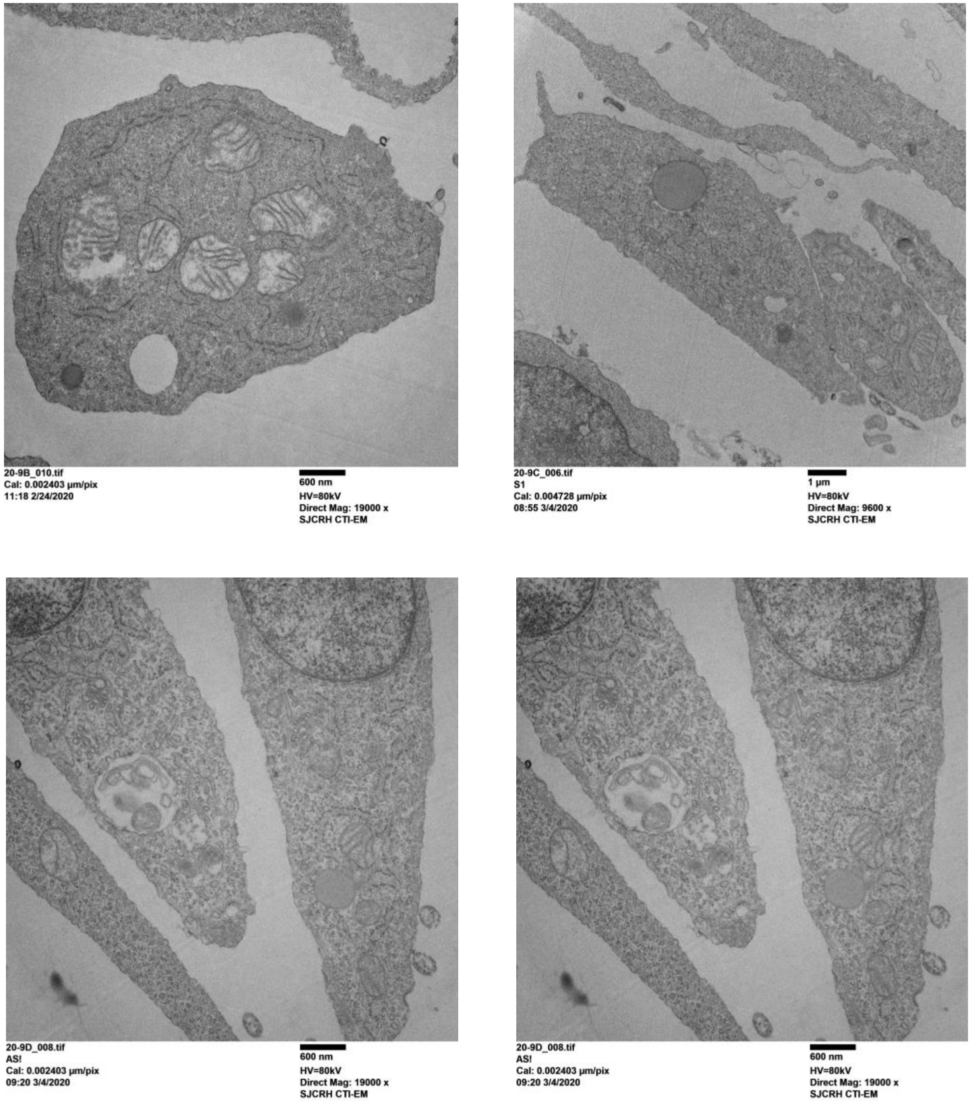

Supplementary Figure 13: Unedited blots for Figure 5a, PPB pulldowns.

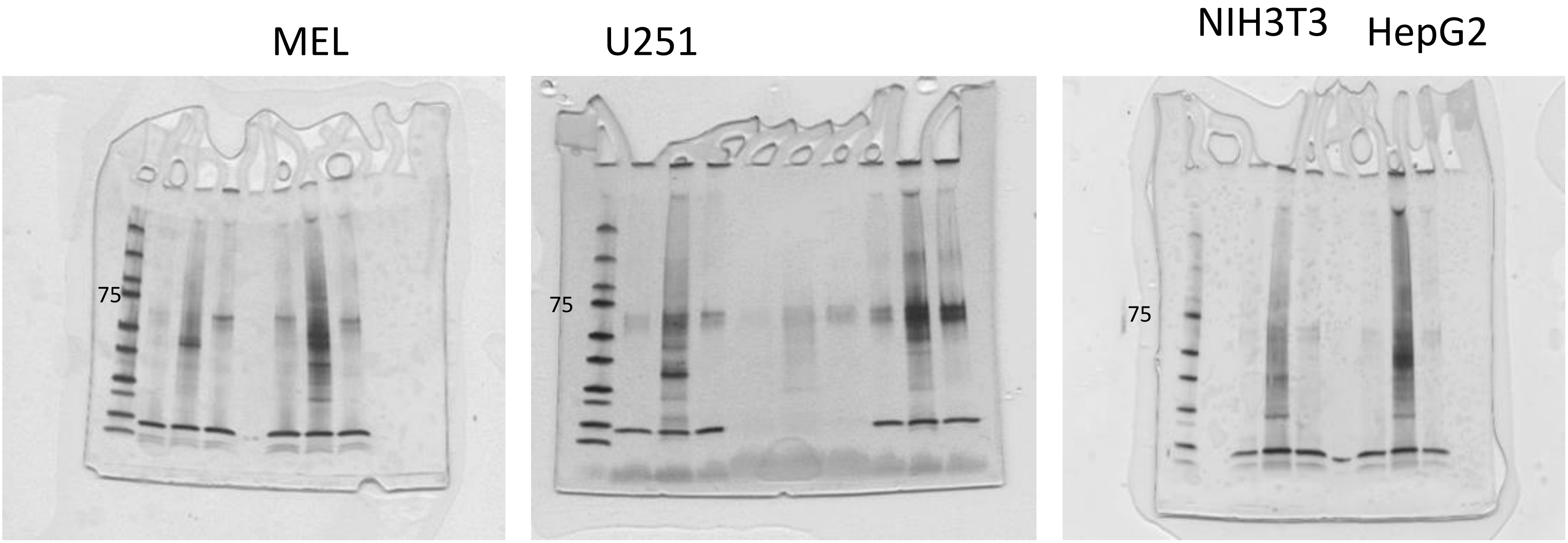

Supplementary Figure 14: Unedited blots for Figure 8d, siRNA knockdown of ACSL4 in NIH3T3.

75

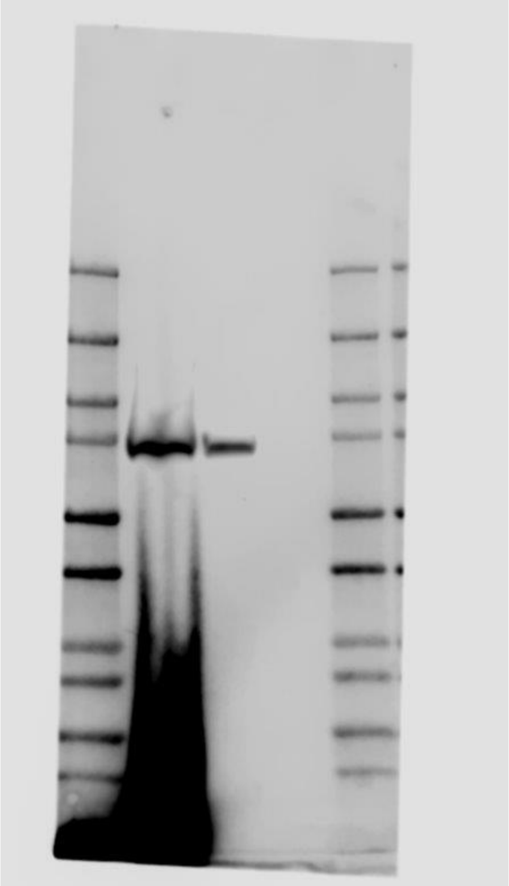

ACSL4

75

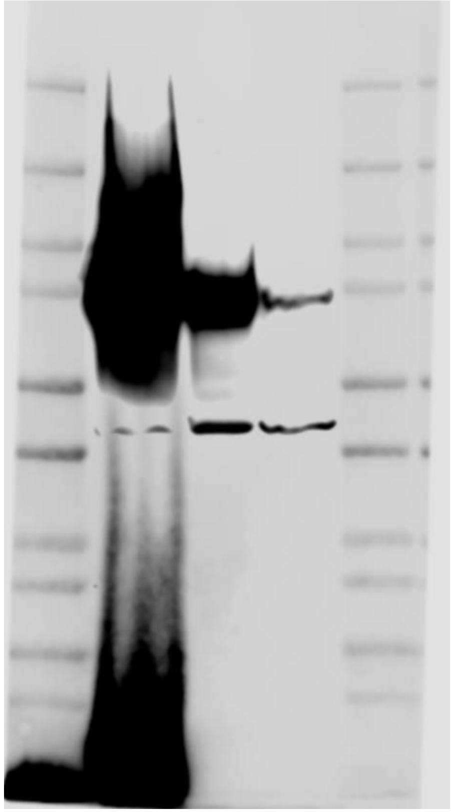

Actin

Supplementary Figure 15: Unedited blots for Figure 8h, siRNA knockdown of H-ferritin in NIH3T3.

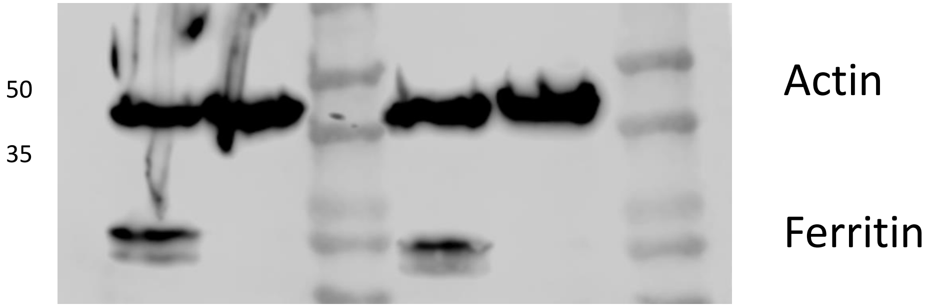

Supplementary Figure 16: Unedited blots for Figure 8j, siRNA knockdown of transferrin receptor in NIH3T3.

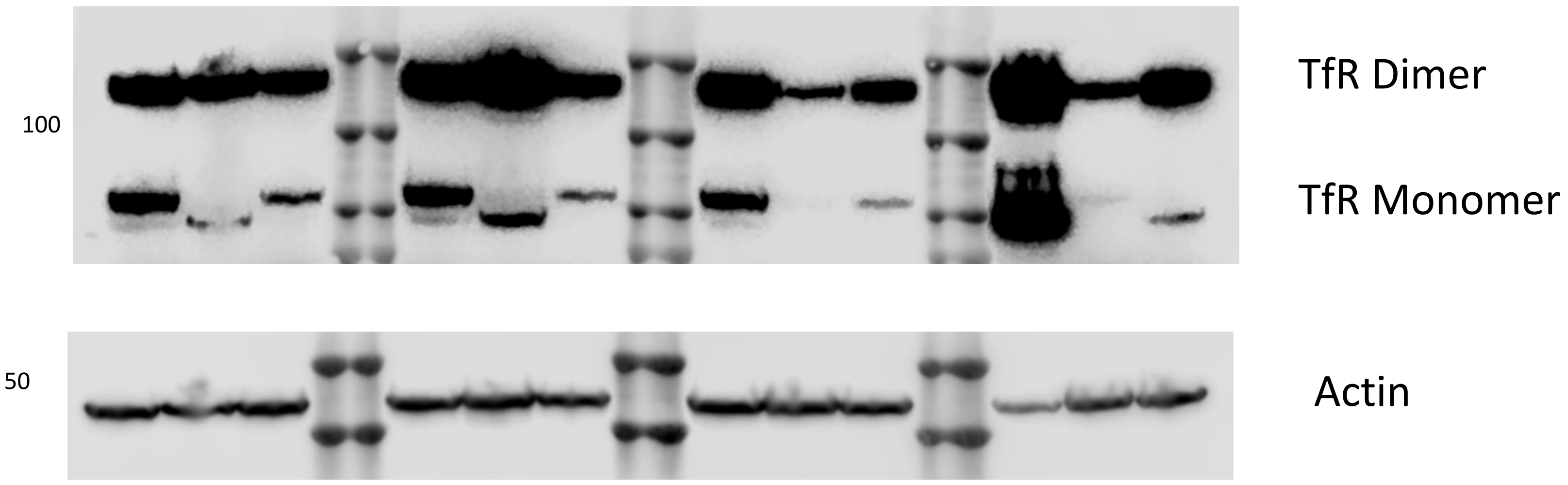

Supplementary Figure 17: Unedited blots for Figure 9a, siRNA knockdown of PRDX1-3 in NIH3T3.

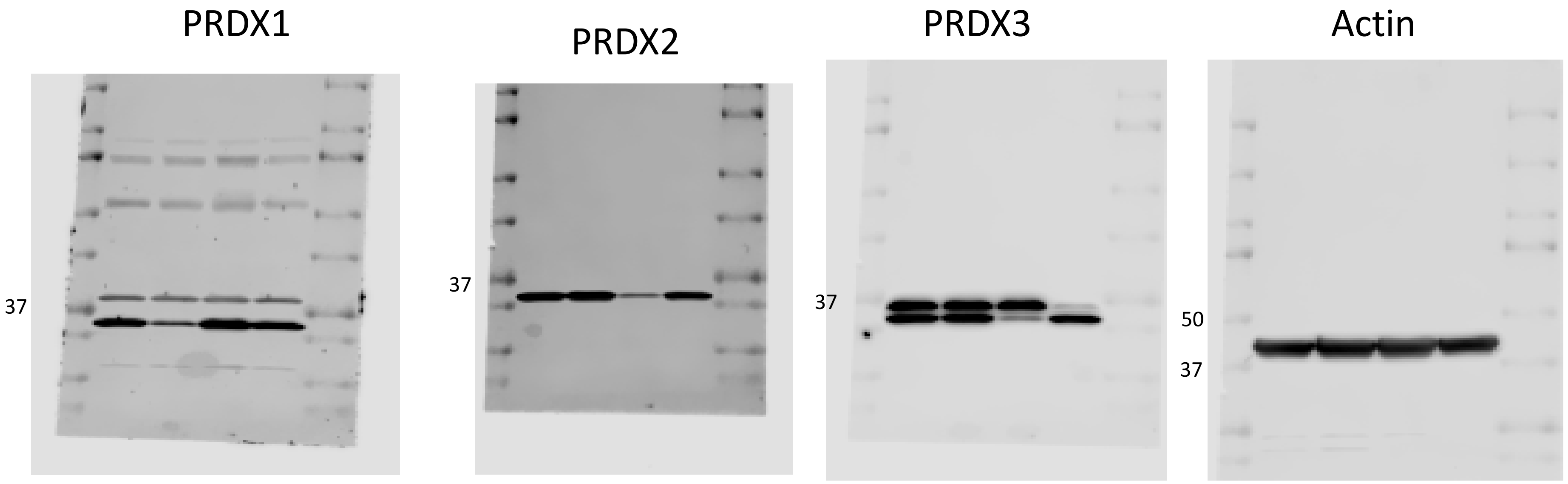

Supplementary Figure 18: Unedited blots for Figure 9c, silver-stain and Western blot of PRD3 pulldown by PPB.

Silver Stain

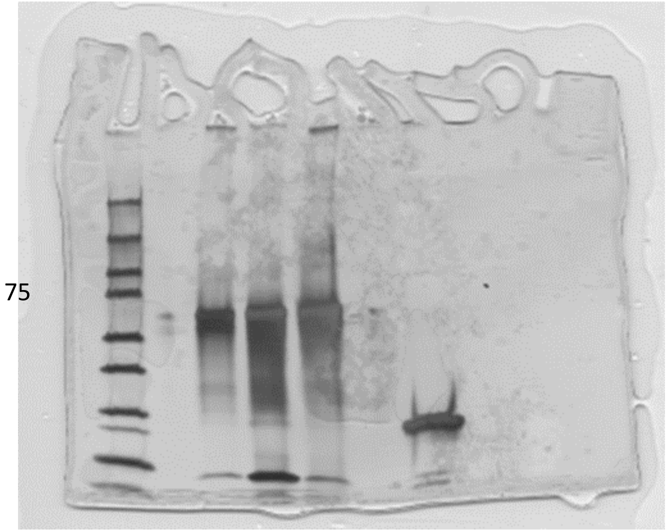

Western Blot

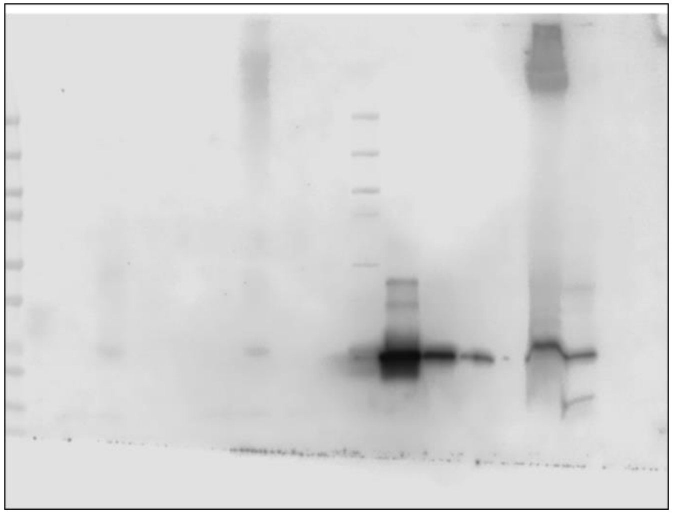

Supplementary Figure 19: Unedited blots for Figure 9j, ACSL4 knockdown in Jurkat.

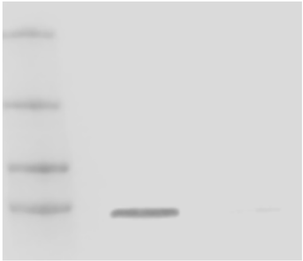

ACSL4

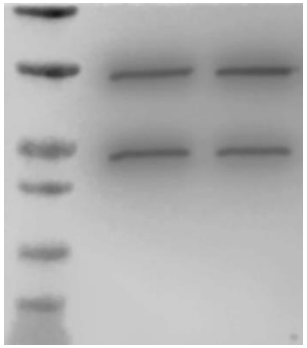

GAPDH

Supplementary Figure 20: Unedited blots for Figure 9k, PRDX3 knockdown in 293T cells.

PRDX3

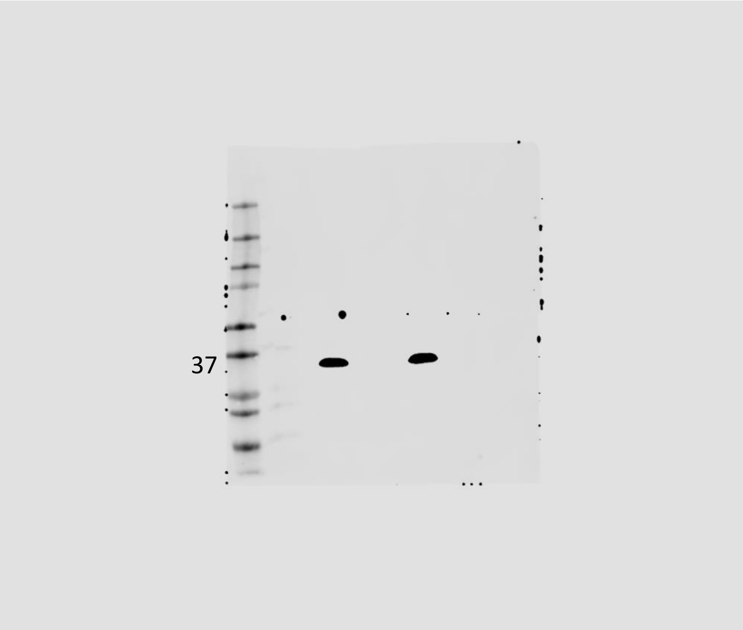

GAPDH

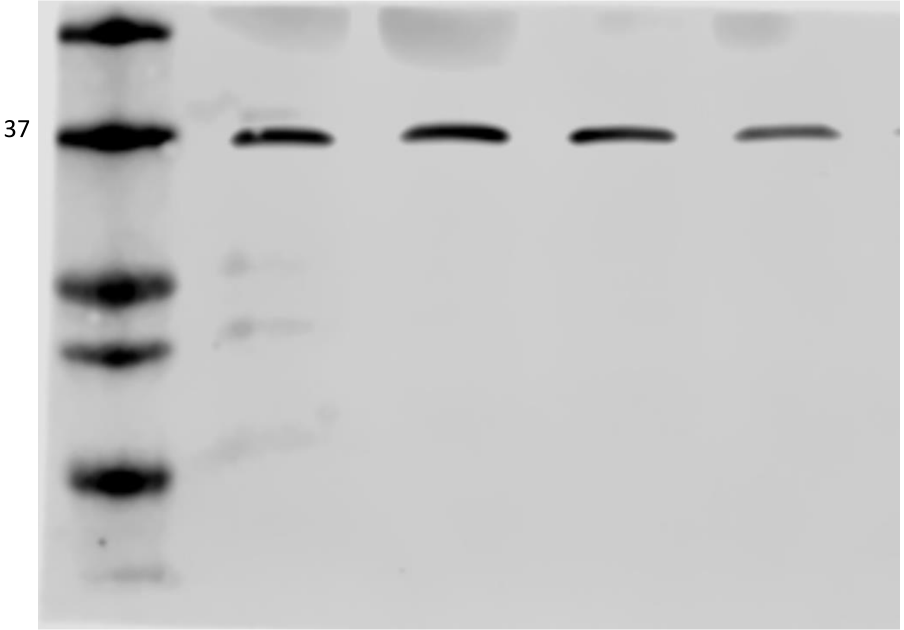

Supplementary Figure 21: Unedited blots for Figure 9I, PRDX3 aggregation in peroxide.

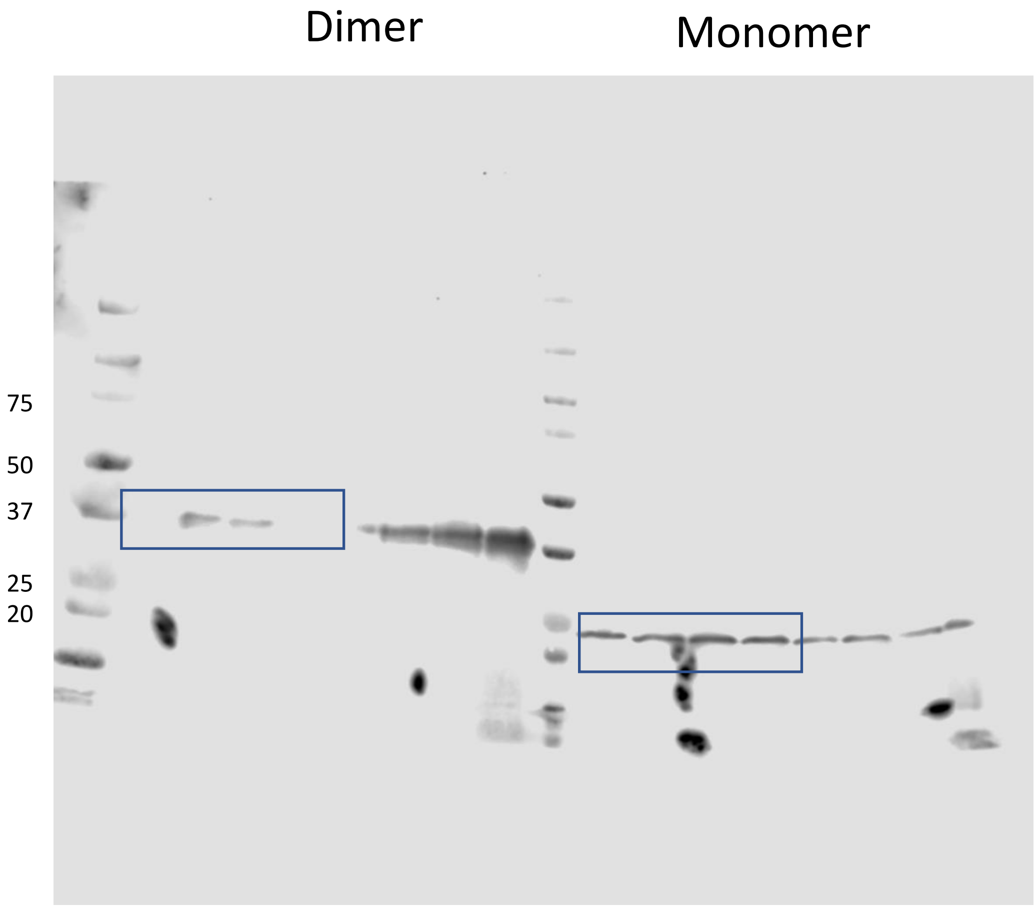

Supplementary Figure 22: Unedited blots for Figure 9m, PRDX3 aggregation in peroxide and PPIX.

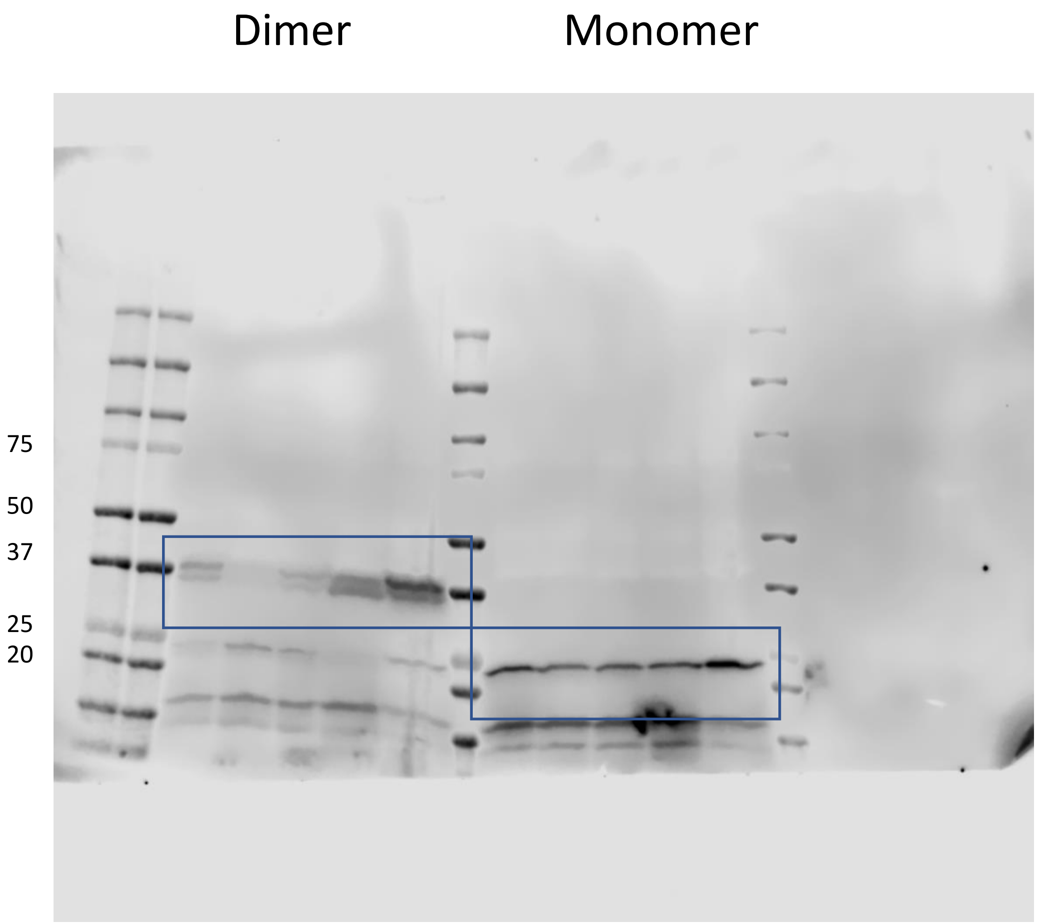

Supplementary Figure 23: Unedited photos for Suplementary Figure 2a, sequence of washes in PPB/PPIX precipitation.

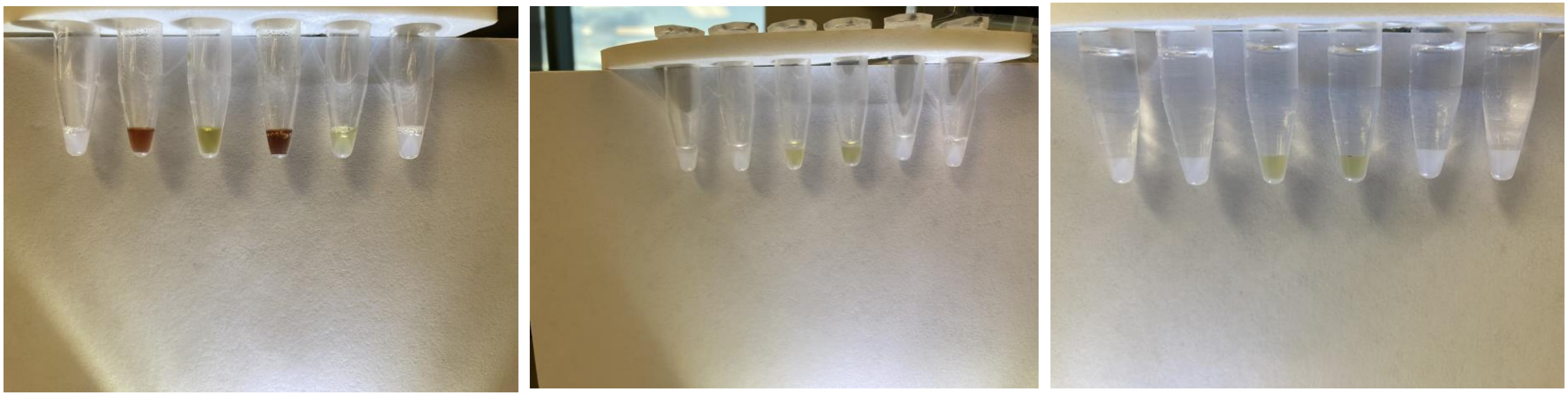

Supplementary Figure 24: Unedited photos for Suplementary Figure 3a, sequence of washes in PPB/PPIX precipitation.

ANT 1/2

150
75
50
37
25

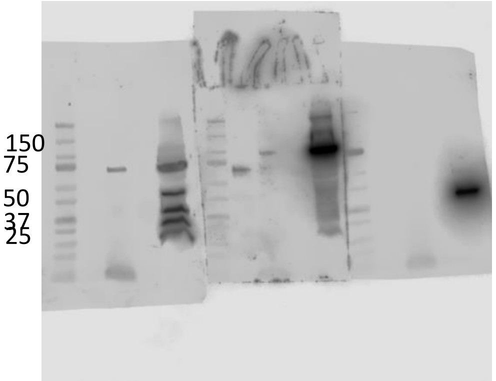

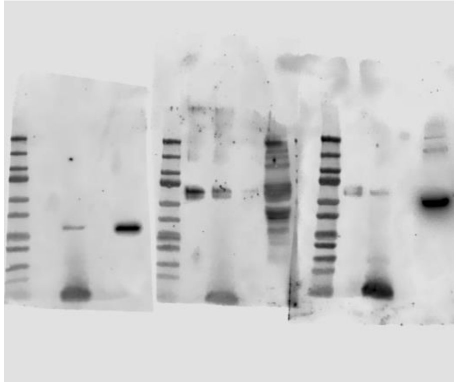

SDHA,B and C

75
50
37
25
15

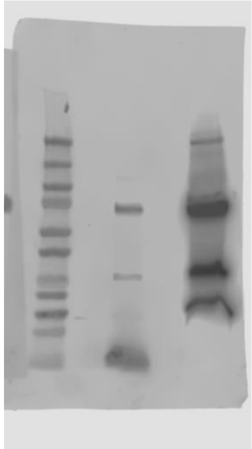

Supplementary Figure 25: Unedited blots for Supplementary Figure 7c, sucrose/ALA effect on surface H-ferritin.

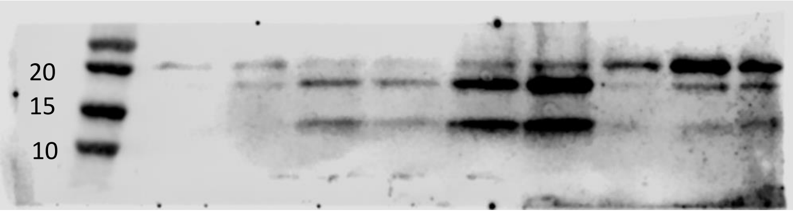

Ferritin

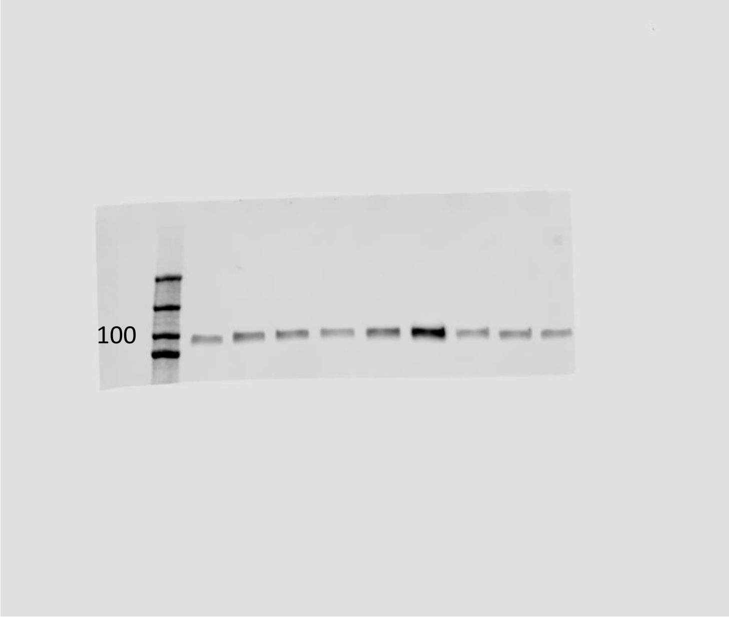

$Na^{+}K^{+}ATPase$
