# Supplementary material for: A PPIX-binding probe facilitates discovery of PPIX-induced cell death modulation by peroxiredoxin
Source: Commun Biol. 2023 Jun 24;6:673. doi: 10.1038/s42003-023-05024-5 (PMC10290680; doi:10.1038/s42003-023-05024-5)
Supplement: Supplementary file 2 — Description of Additional Supplementary Files [file 42003_2023_5024_MOESM2_ESM.pdf]

Description of Additional Supplementary Files

File name: Supplementary Data 1

Description: Candidate PPB binding proteins and full-list of identified proteins via TMT.

File name: Supplementary Data 2

Description: PPIX-binding protein pathway analysis, KEGG, Keyword search, etc.

File name: Supplementary Data 3

Description: Proteins composing individual clusters

File name: Supplementary Data 4

Description: Database searches for redox proteins compared to PPB-binding results.

File name: Supplementary Data 5

Description: Cluster pathway analysis and subcellular localization data.

File name: Supplementary Data 6

Description: Candidate PPB binding proteins identified through Saint Analysis.

File name: Supplementary Data 7

Description: Numerical source data for all charts and graphs
