# Supplementary material for: A PPIX-binding probe facilitates discovery of PPIX-induced cell death modulation by peroxiredoxin
Source: Commun Biol. 2023 Jun 24;6:673. doi: 10.1038/s42003-023-05024-5 (PMC10290680; doi:10.1038/s42003-023-05024-5)
Supplement: Supplementary file 10 — Reporting Summary [file 42003_2023_5024_MOESM10_ESM.pdf]

nature portfolio

Corresponding author(s): John D. Schuetz

Last updated by author(s): 5/22/2023

Reporting Summary

Nature Portfolio wishes to improve the reproducibility of the work that we publish. This form provides structure for consistency and transparency in reporting. For further information on Nature Portfolio policies, see our Editorial Policies and the Editorial Policy Checklist.

Statistics

For all statistical analyses, confirm that the following items are present in the figure legend, table legend, main text, or Methods section.

n/a	Confirmed	
☐	☒	The exact sample size (*n*) for each experimental group/condition, given as a discrete number and unit of measurement
☐	☒	A statement on whether measurements were taken from distinct samples or whether the same sample was measured repeatedly
☐	☒	The statistical test(s) used AND whether they are one- or two-sided<br>*Only common tests should be described solely by name; describe more complex techniques in the Methods section.*
☐	☒	A description of all covariates tested
☐	☒	A description of any assumptions or corrections, such as tests of normality and adjustment for multiple comparisons
☐	☒	A full description of the statistical parameters including central tendency (e.g. means) or other basic estimates (e.g. regression coefficient) AND variation (e.g. standard deviation) or associated estimates of uncertainty (e.g. confidence intervals)
☒	☐	For null hypothesis testing, the test statistic (e.g. *F*, *t*, *r*) with confidence intervals, effect sizes, degrees of freedom and *P* value noted<br>*Give P values as exact values whenever suitable.*
☒	☐	For Bayesian analysis, information on the choice of priors and Markov chain Monte Carlo settings
☒	☐	For hierarchical and complex designs, identification of the appropriate level for tests and full reporting of outcomes
☒	☐	Estimates of effect sizes (e.g. Cohen's *d*, Pearson's *r*), indicating how they were calculated

Our web collection on statistics for biologists contains articles on many of the points above.

Software and code

Policy information about availability of computer code

Data collection	Shimadzu Class VP 7.0, Gen 5 3.05.11
Data analysis	GraphPad Prism (version 9.02 for Windows), Image Studio Line (I-COR version 5.2), JUMP for mass spectrometry, FlowJo v10.8

For manuscripts utilizing custom algorithms or software that are central to the research but not yet described in published literature, software must be made available to editors and reviewers. We strongly encourage code deposition in a community repository (e.g. GitHub). See the Nature Portfolio guidelines for submitting code & software for further information.

Data

Policy information about availability of data

All manuscripts must include a data availability statement. This statement should provide the following information, where applicable:

- Accession codes, unique identifiers, or web links for publicly available datasets
- A description of any restrictions on data availability
- For clinical datasets or third party data, please ensure that the statement adheres to our policy

Proteomic data will be available through the Dryad data depository via doi:10.5061/dryad.mkkwh712t. Numerical source data for all charts and graphs is available in Supplementary Data Table 1. Original images for figures may be found, as Supplementary Figures 12-25, in the Supplementary Information file. Any other data will be provided upon request of the corresponding author.

Research involving human participants, their data, or biological material

Policy information about studies with human participants or human data. See also policy information about sex, gender (identity/presentation), and sexual orientation and race, ethnicity and racism.

Reporting on sex and gender	N/A
Reporting on race, ethnicity, or other socially relevant groupings	N/A
Population characteristics	N/A
Recruitment	N/A
Ethics oversight	N/A

Note that full information on the approval of the study protocol must also be provided in the manuscript.

Field-specific reporting

Please select the one below that is the best fit for your research. If you are not sure, read the appropriate sections before making your selection.

☒ Life sciences ☐ Behavioural & social sciences ☐ Ecological, evolutionary & environmental sciences

For a reference copy of the document with all sections, see nature.com/documents/nr-reporting-summary-flat.pdf

Life sciences study design

All studies must disclose on these points even when the disclosure is negative.

Sample size	Sample size was chosen based on the nature of each experiment. Analysis of electron microscopy used >70 distinct fields for each condition for analysis. For most other experiments three biological independent experiments were performed with 2-5 independent replicates per data point.
Data exclusions	Data was not selectively excluded from experiments. When technical issues rendered data unusable from an experiment, all data was excluded from analysis.
Replication	Results of all replicate experiments were fundamentally in agreement.
Randomization	N/A
Blinding	Blind assessment was used for the collection of all subjective data.

Reporting for specific materials, systems and methods

We require information from authors about some types of materials, experimental systems and methods used in many studies. Here, indicate whether each material, system or method listed is relevant to your study. If you are not sure if a list item applies to your research, read the appropriate section before selecting a response.

Materials & experimental systems

n/a	Involved in the study	
☐	☒	Antibodies
☐	☒	Eukaryotic cell lines
☒	☐	Palaeontology and archaeology
☒	☐	Animals and other organisms
☒	☐	Clinical data
☒	☐	Dual use research of concern
☒	☐	Plants

Methods

n/a	Involved in the study	
☒	☐	ChIP-seq
☒	☐	Flow cytometry
☒	☐	MRI-based neuroimaging

Antibodies

Antibodies used	

Ferritin, Abcam, AB75973
Na+K+ATPase, Novus, NB300-146SS
Prdx1, Invitrogen, LF-MA0073
Prdx2, Invitrogen, LF-MAO144
Prdx3, Abnova, MAB2725
Transferrin Receptor, Zymed 13-6800

Validation	All commercial antibodies were validated according to the companies for the species for which it was employed.

Eukaryotic cell lines

Policy information about cell lines and Sex and Gender in Research

Cell line source(s)	NIH3T3, Jurkat, 293T, MEL, HepG2, U251.
Authentication	None Attempted.
Mycoplasma contamination	Tested for mycoplasma.
Commonly misidentified lines (See ICLAC register)	None.
